# Analysis of Daily Ambient Temperature and Firearm Violence in 100 US Cities

**DOI:** 10.1001/jamanetworkopen.2022.47207

**Published:** 2022-12-16

**Authors:** Vivian H. Lyons, Emma L. Gause, Keith R. Spangler, Gregory A. Wellenius, Jonathan Jay

**Affiliations:** 1 Social Development Research Group, School of Social Work, University of Washington, Seattle; 2 Allies in Healthier Systems for Health & Abundance in Youth, Department of Psychiatry, University of Washington, Seattle; 3 Firearm Injury & Policy Research Program, Harborview Injury Prevention & Research Center, University of Washington, Seattle; 4 Department of Environmental Health, Boston University School of Public Health, Boston, Massachusetts; 5 Department of Community Health Sciences, Boston University School of Public Health, Boston, Massachusetts

## Abstract

**Importance:**

Firearm violence is a leading public health crisis in the US. Understanding whether and how ambient temperature is associated with firearm violence may identify new avenues for prevention and intervention.

**Objective:**

To estimate the overall and regional association between hotter temperatures and higher risk of firearm violence in the US.

**Design, Setting, and Participants:**

This cross-sectional study used distributed lag nonlinear models, controlling for seasonality and long-term time trends by city and pooled results overall and by climate region. The most populous cities in the US with the highest number of assault-related firearm incidence (ie, shootings) from 2015 to 2020 were analyzed. Data analysis was performed from October 2021 to June 2022.

**Exposures:**

Maximum daily temperature by city.

**Main Outcomes and Measures:**

The primary outcome was the number of assault-related firearm shootings by city.

**Results:**

A total of 116 511 shootings in 100 cities were included in this analysis. The pooled analysis estimated that 6.85% (95% CI, 6.09%-7.46%) of all shootings were attributable to days hotter than city-specific median temperatures. This equates to 7973 total shootings (95% CI, 7092-8688 total shootings) across the 100 cities over the 6-year study period, although the number of total persons injured or killed would be higher. Estimated risk of firearm incidents increased almost monotonically with higher temperatures, with a local peak at the 84th percentile of the temperature range corresponding to a relative risk of 1.17 (95% CI, 1.12-1.21) compared with the median temperature. However, even moderately hot temperatures were associated with higher risk of shootings. Although significant, there was low heterogeneity between cities (*I*^2^ = 11.7%; Cochran *Q* test, *P* = .02), indicating regional or climate-specific variation in the daily temperature and incident shootings relationship.

**Conclusions and Relevance:**

These findings underscore the importance of heat adaptation strategies broadly throughout the year to reduce shootings, rather than focusing on only the hottest days.

## Key Points

**Question:**

Are higher temperatures associated with increased risk of a firearm shooting?

**Findings:**

In this cross-sectional analysis of the 100 cities with largest burden of firearm shootings in the US, 6.85% of all shootings were associated with above-average temperatures.

**Meaning:**

These findings indicate a need for heat adaptation strategies for mitigation of risk of firearm shootings.

## Teaser

This cross-sectional study estimates the overall and regional association between hotter temperatures and higher risk of firearm violence in the US.

## Introduction

Interpersonal firearm violence is a public health crisis in the US. Although the rate of firearm homicides against persons older than 12 years reached historical lows in the early 2010s (4.0 homicides per 100 000 in 2014), they have been increasing since then (6.8 homicides per 100 000 in 2020).^1,2^ As of 2020, firearms are the leading cause of death for children and adolescents and account for 77% of all homicides in the US.^1,3^ These interpersonal firearm deaths (ie, homicides) are almost twice as common in urban compared with rural areas.^4^ For every individual who dies from an assault-related firearm injury, approximately 2.4 individuals survive with injuries requiring emergency department care, and they face substantial emotional, physical, and economic challenges.^5,6^ The most common precipitating circumstance is disputes between individuals (41% of cases).^7^ Given the scope of this public health crisis and recent increasing rates, it is vital to understand the contextual factors that may influence these outcomes.

Climate change may be one of these influences. Conceptually, warmer temperatures increase stress hormones in the nervous system, which could increase individual potential for violence (ie, the temperature-aggression theory).^8^ Warmer temperatures could also increase social contacts and time spent outdoors, increasing the potential for interpersonal conflicts (ie, the routine activities theory).^9^ These phenomena would produce both seasonality (ie, higher violence in warmer months) and greater violence on unseasonably warm days. Seasonality is well-established,^10,11,12,13^ but also potentially attributable to seasonal variation in factors unrelated to weather (eg, school calendars); by contrast, the effects of unseasonably warm days are more likely attributable to temperature alone. Our analysis adds to existing literature in this area by explicitly adjusting for seasonality to assess the association of heat with firearm incidents beyond what would be expected for the time of year.

Past studies have linked warmer temperatures and higher incidence of gun violence. A 2020 study^11^ found shootings in Chicago to be more likely to occur on warm days. A Baltimore study^13^ found an association between daily temperature and shootings, whereas a separate Chicago study^14^ found day of the week and daily temperature to be associated with firearm injuries requiring medical treatment. However, these studies have not distinguished between seasonal vs daily temperature variation, thus narrowing their interpretability. Moreover, they have had limited geographic scope (eg, single city) and do not assess for regional variation, limiting their generalizability to regions with different climate patterns.

The current cross-sectional, time-series study aims to estimate the association of higher daily temperatures with urban firearm violence on a national level, while assessing for variation across cities and regions. This goal is important because urban heat is predictable and, to some degree, modifiable. Cities can use climate adaptation strategies to mitigate the effects of daily temperature through changes to the built environment.^15,16^ If there is an association between daily temperature and firearm violence, implementation of climate adaptation strategies may be associated with reduced firearm violence on unseasonably warm days.

## Methods

### Exposure

We estimated maximum daily temperatures by city for each day from 2015 to 2020 using hourly, 2 m, gridded air temperatures from the North American Land Data Assimilation System Phase 2.^17,18^ Daily city-specific maximum temperatures were calculated as the population-weighted mean of the maximum daily temperature for each pixel within the city’s geographical boundary. Temperatures were converted to city-specific percentiles to allow for relative comparisons between cities with different temperature distributions and to acknowledge the impact of acclimatization on individual experience of heat. Because all data used in the study were publicly available, the Boston University institutional review board waived review as non–human participants research, and informed consent was not required, in accordance with 45 CFR §46. This study follows the Strengthening the Reporting of Observational Studies in Epidemiology (STROBE) reporting guidelines for cross-sectional studies.

### Outcome

Our outcome of interest was the number of firearm incidents with at least 1 person injured or killed (henceforth referred to as shootings) by city, captured using Gun Violence Archive (GVA) data.^19^ We used incident-level counts because the number of specific individuals injured or killed in a single shooting may vary widely between localities owing to availability of high-capacity magazines or other factors influenced by local policies.

Our study was limited to officer-involved, unintentional, and assault-related shootings. It did not include suicides or intentional self-harm injuries because these are not available from GVA for analysis. The majority are likely assault related: a test case validation using US Census tract level counts of GVA assault-related shootings correlated at 0.98 with Philadelphia police data for assault-related shootings.^20^

### Study Setting

Using GVA data, we selected the 100 cities with the greatest counts of shootings from 2015 to 2020 with high burdens of firearm violence to support day-level analyses. Cities were selected from among the 500 most populous cities in the US.^21^ The shootings in these 100 cities represent 83% of all shootings reported during this period in the GVA data.

### Statistical Analysis

 Data analysis was performed from October 2021 to June 2022. We used distributed lag nonlinear models (DLNMs) to quantify the association between temperature and shootings in each city. This modeling approach allows for flexibility in modeling nonlinear associations between exposure and outcome, the estimation of a time-lagged association of temperature on shootings, and also controls for seasonality and long-term time trends.^22^

In the first stage of the analysis, we fit a quasi-Poisson DLNM individually in each of the 100 cities included in the analysis, with maximum daily temperature as the exposure and the daily count of shootings as the main outcome of interest, adjusting for day of the week. Individual city analyses were fit using city-specific temperature percentiles to allow conversion back to an absolute temperature to better understand regional or city-specific risk in their local context. Seasonal patterns and year-over-year trends for each city were controlled for by including cubic basis splines with 7 knots per study year (eFigure 1 in Supplement 1).^23^

DLNMs estimated both the daily and lagged association between temperature and shootings occurring over a specified lagged period.^22^ The temperature-shootings association was modeled using quadratic splines with knots at the 10th, 75th, and 90th percentiles of each city-specific temperature range to allow for a nonlinear association between temperature and shootings, as well as changes in direction at temperature extremes.^24^ We fit a lag period of up to 7 days and found a 0-day lag to be the best fit (eFigure 2 in Supplement 1). Sensitivity analyses were conducted testing different numbers and placements of knots and days of lag to assess the results’ sensitivity to model specification (eFigure 3 and eFigure 4 in Supplement 1), or a potential displacement effect (eTable 1 and eFigure 5 in Supplement 1).

**Figure 1.**
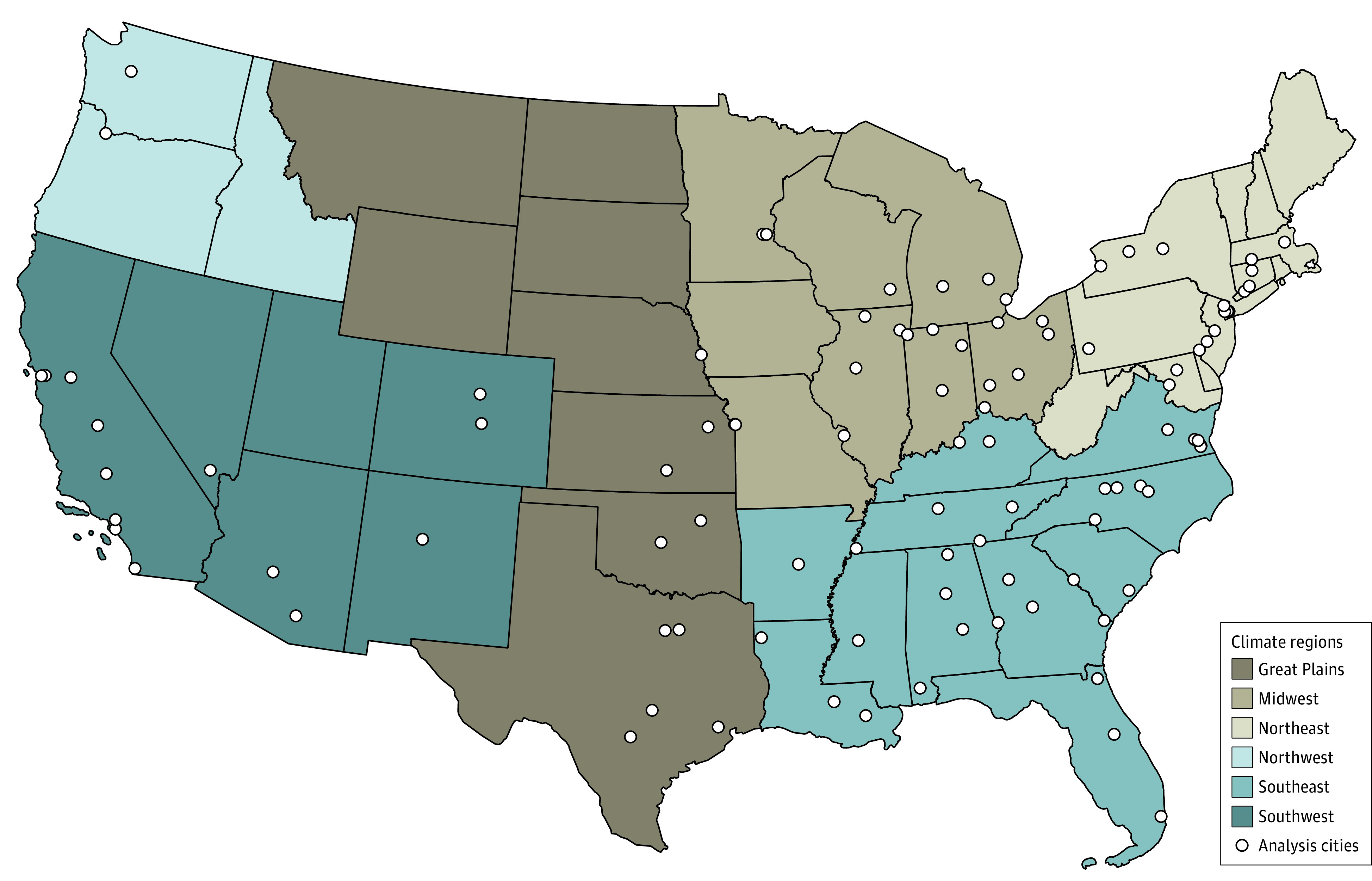
Geographic Distribution of the 100 Analysis Cities by Climate Region This map shows states categorized according to their climate region (from the National Climate Assessment). The white dots represent the location of the 100 cities that were included in this analysis.

We then fit a multivariate metaregression model to the city-specific results, which incorporates the covariance between cities, to pool the estimates across the 100 cities with city-specific mean temperature and temperature range included as metavariables.^25^ A Wald test was used to test whether the metavariables significantly modified the temperature and firearm association. Heterogeneity in the temperature-shootings association between cities was assessed using Cochran *Q* test and quantified using the *I*^2^ statistic to determine whether pooling across cities in our analysis was appropriate. Stratification by climate region was conducted to investigate potential regional differences (Table and Figure 1).^25^ Following a Bayesian approach, we increased precision for cities with small numbers or high variance by using the results of the pooled metaregression were used to estimate the best unbiased linear predictors of the association in each city.^26^

**Table.** Overall and Regionally Stratified Pooled Associations of Daily Temperature and Shootings Association^a^

Region	Cities, No.	Attributable risk, % (95% CI)			Shootings, No.	Median temperature, °C
		All temperatures	Moderate heat	Extreme heat		
Overall model	100	6.85 (6.09 to 7.46)	5.00 (4.44 to 5.43)	1.86 (1.58 to 2.05)	116 511	NA
Regional estimates						
Great Plains	11	4.12 (2.21 to 5.70)	3.37 (2.13 to 4.45)	0.75 (−0.02 to 1.32)	11 175	25.3
Midwest	21	9.51 (8.46 to 10.50)	6.79 (5.95 to 7.58)	2.73 (2.36 to 3.02)	39 714	17.2
Northeast	18	9.54 (6.55 to 11.90)	6.96 (5.01 to 8.38)	2.60 (1.49 to 3.40)	25 970	16.9
Northwest^b^	2	NA	NA	NA	964	15.9
Southeast	34	2.98 (1.95 to 3.70)	2.38 (1.63 to 2.96)	0.61 (0.17 to 0.92)	29 389	25.1
Southwest	14	0.48 (−1.58 to 2.17)	0.02 (−1.46 to 1.17)	0.46 (−0.36 to 1.07)	9299	22.2

Abbreviation: NA, not applicable.

^a^ The table shows the results of the overall heat and firearm associations pooled across the 100 analysis cities, as well as the results of the sensitivity analysis stratified by climate region. Median temperatures were calculated from within each climate region to show some of the potential heterogeneity in temperatures experienced in cities within each region. All models were implemented with 0 lag days.

^b^ There were too few cities to reliably estimate overall climate region estimates.

The main results measures were the proportion and absolute number of shootings attributable to temperatures above the city-level median. These measures were calculated for each city, pooled by region and across the full sample, and then decomposed according to moderate and extreme heat components. We defined moderate and extreme heat as temperatures from the median to 90th percentile and 90th to 100th percentile, respectively, of the city-specific temperature range. The city-specific median temperature was chosen to represent the counterfactual scenario for estimating the relative risks used in this calculation: the excess risk of firearm incidents at each temperature percentile was estimated by comparing it with the city’s median temperature. The attributable fraction was calculated by summing the daily heat-attributable shooting estimates across the time-series for each city and then dividing by the total number of shootings in each city over the study period, following the established method for calculating attributable risk (AR) estimates in complex, nonlinear time-series models developed by Gasparrini et al.^27^ Ninety-five percent confidence intervals were estimated using a Monte-Carlo simulation, assuming a multivariate normal distribution of the best unbiased linear predictors. The daily shootings that were attributable to hot days, beyond what would be expected for the season and day of the week, were also graphed to visualize trends throughout the year.

Additional sensitivity analyses were conducted to investigate whether the inclusion of 2020 data, a year marked both by COVID-19 pandemic disruptions to daily life as well as civil movements in protest of police brutality and systemic racism, might affect the association between heat and shootings in the primary analysis. Beginning in 2020, there were higher than average rates of firearm violence (eFigure 1 in Supplement 1),^28^ but we hypothesized this level change would not modify the association between heat and shootings, particularly after adjusting for the seasonal trend in this year. First, a pandemic indicator was included in the model for days in March 2020 and beyond. Second, the analysis was repeated, restricting the data years to remove 2020 from the analysis. Analyses were performed with R statistical software version 4.0.5 (R Project for Statistical Computing). See the eAppendix in Supplement 1 for additional details on statistical software and packages.

## Results

There were 116 511 shootings recorded across the 100 cities over the 6-year study period from 2015 to 2020, with a range of 16 to 202 shootings across all cities on any given day. The number of shootings tended to be higher in the summer, and 2020 experienced a spike in shootings compared with previous years (eFigure 1 in Supplement 1).

**Figure 2.**
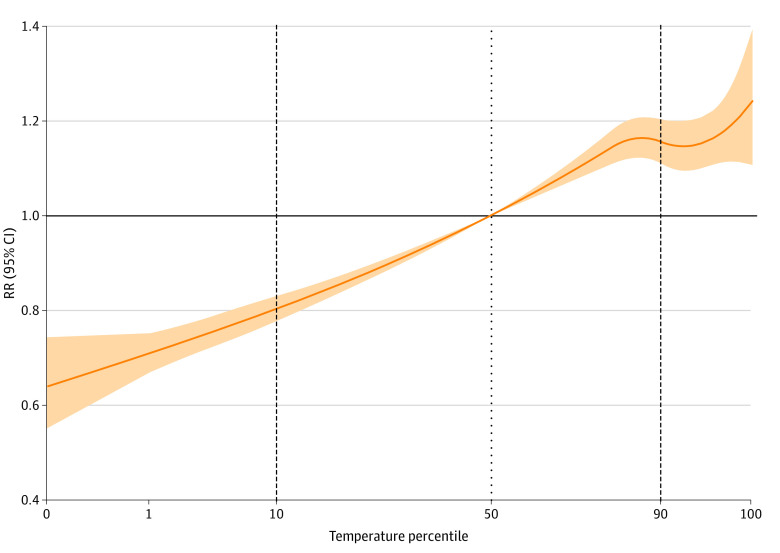
Cumulative Association of Daily Temperature and Relative Risk (RR) of Shootings Across 100 Cities From 2015-2020 This graph depicts the overall pooled association between maximum daily temperature and shootings across 100 cities, with the lag duration specified as 0 days. The median temperature percentile is used as the counterfactual scenario to estimate RRs, with 95% CIs depicted by shaded areas. Dashed lines are drawn at the 10th and 90th percentiles of the temperature range to mark extreme low and extreme high temperatures. Moderate heat is classified as temperatures between the city-specific 50th and 90th percentiles, whereas extreme heat is classified as temperatures greater than the 90th percentile.

The estimated association between temperature and shootings increased almost monotonically across the temperature range, with a local peak at the 84th percentile of the temperature range with a relative risk of 1.17 (95% CI, 1.12-1.21) compared with the median. Although the estimated relative risk decreased slightly after that, it remained elevated throughout the extreme heat temperature range (Figure 2) and may have increased again around the 99th percentile, although estimation at that range is unstable. The temperature at which this peak occurred differed across cities, but for the majority of cities it fell between 84 °F (29 °C) and 90 °F (32 °C).

From the pooled analysis, we estimate that 6.85% (95% CI, 6.09% to 7.46%) of all shootings were attributable to hot days. This equates to 7973 total shootings (95% CI, 7092 to 8688 total shootings) across the 100 cities over the 6-year study period, although the number of total persons injured or killed would be higher; each shooting during this period resulted in a mean (SD) of 1.25 (0.73) persons injured or killed (range, 1-103 individuals per shooting). A greater proportion of shootings was attributable to moderate temperatures (5.00%; 95% CI, 4.44% to 5.43%) vs extreme heat days (1.86%; 95% CI, 1.58% to 2.05%), although risk was generally greater with higher temperatures (Table). Although it was significant, there was low heterogeneity between cities in the pooled meta-analysis (*I*^2^ = 11.7%; Cochran *Q* test, *P* = .02), indicating 11.7% of the variability in the pooled relative risk estimates is attributable to true heterogeneity between cities. This finding suggests that although our 100 analysis cities share a common general association between daily temperature percentile and firearm incidents, there remains some variability unique to each local context. This could be due to regional or climate-specific variation in the association between daily temperature and shootings. Average city temperature was found to significantly modify the temperature and firearm shooting association in the meta-analysis, whereas temperature range was not. The highest AR between daily temperature and shootings was found in the Northeast (AR, 9.54%; 95% CI, 6.55% to 11.90%) and Midwest (AR, 9.51%; 95% CI, 8.46% to 10.50%), areas with lower median regional temperatures than the Southwest, which had the smallest temperature-shootings AR (AR, 0.48%; 95% CI, −1.58% to 2.17%) (Table). We could not estimate a pooled association for the Northwest region because only 2 cities in that region met inclusion criteria and were included in this analysis, but the city-specific results for these 2 cities can be found in eTable 2 in Supplement 1 (Seattle, Washington, AR, 12.00%; 95% CI, 4.34% to 17.75%; Portland, Oregon, AR, 8.71%; 95% CI, 1.57% to 14.7%).

**Figure 3.**
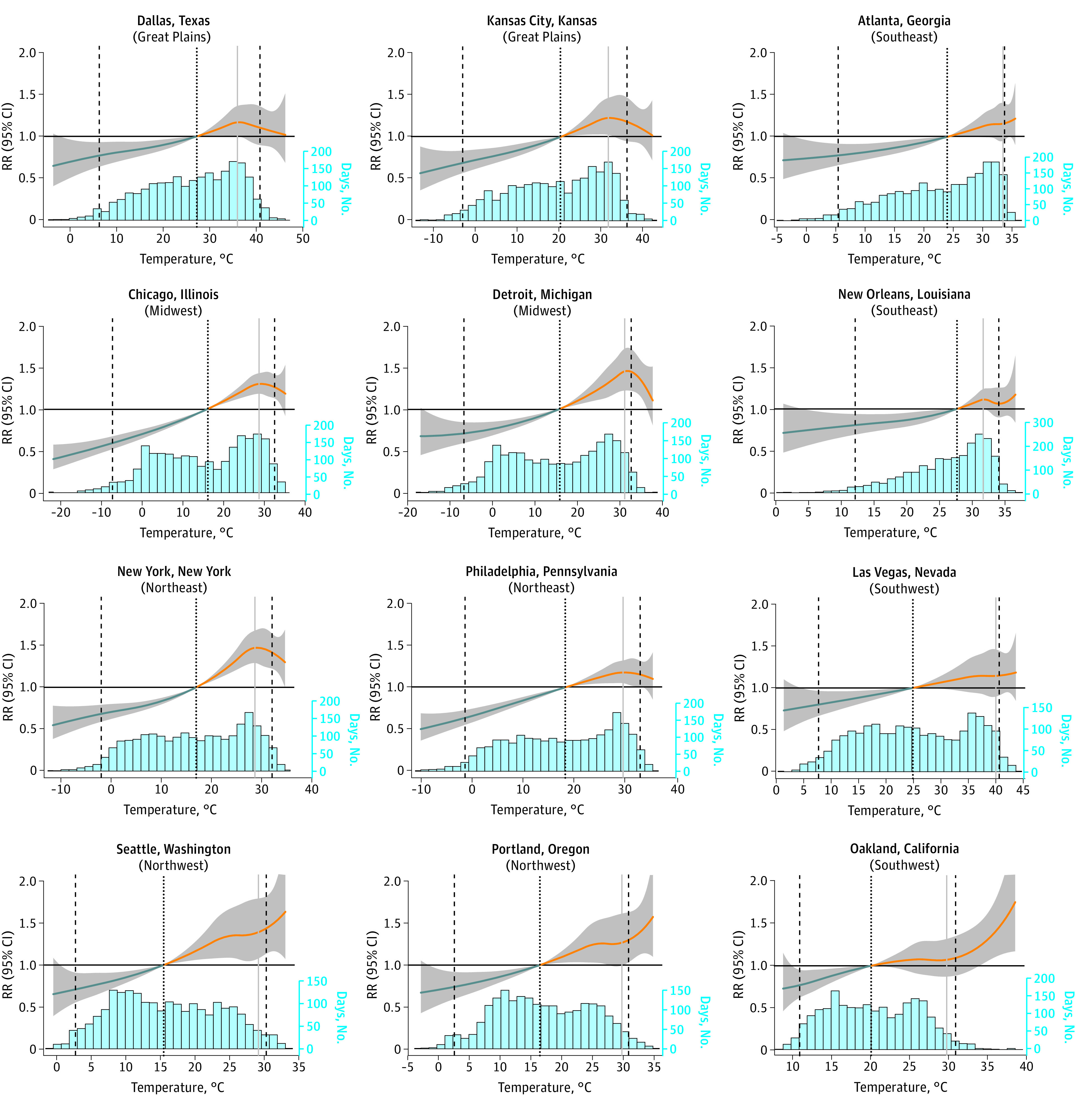
Overall Association of Temperature and Shootings in 12 Cities Across Climate Regions These graphs depict the city-specific associations between maximum daily temperature and shootings across the temperature range from the distributed lag nonlinear model specified with a 0-day lag period. Histograms below the graphs show the range and frequency of temperature days for each city. Two cities from each climate region were selected to display. The median temperature of each city was used as the counterfactual scenario for the estimation of relative risks (marked with the black dotted vertical line). Temperatures above the median are considered hot days (orange lines). The 2.5th and 97.5th percentiles of the city-specific temperature range are marked with black dashed vertical lines. The temperature percentile with the highest relative risk is marked with a vertical gray line (constrained to be within the inner 95% of temperature percentiles to avoid potential spurious results due to sparsity at the tails of the temperature range). City-specific association graphs for all 100 cities can be found in eFigure 7 in Supplement 1.

**Figure 4.**
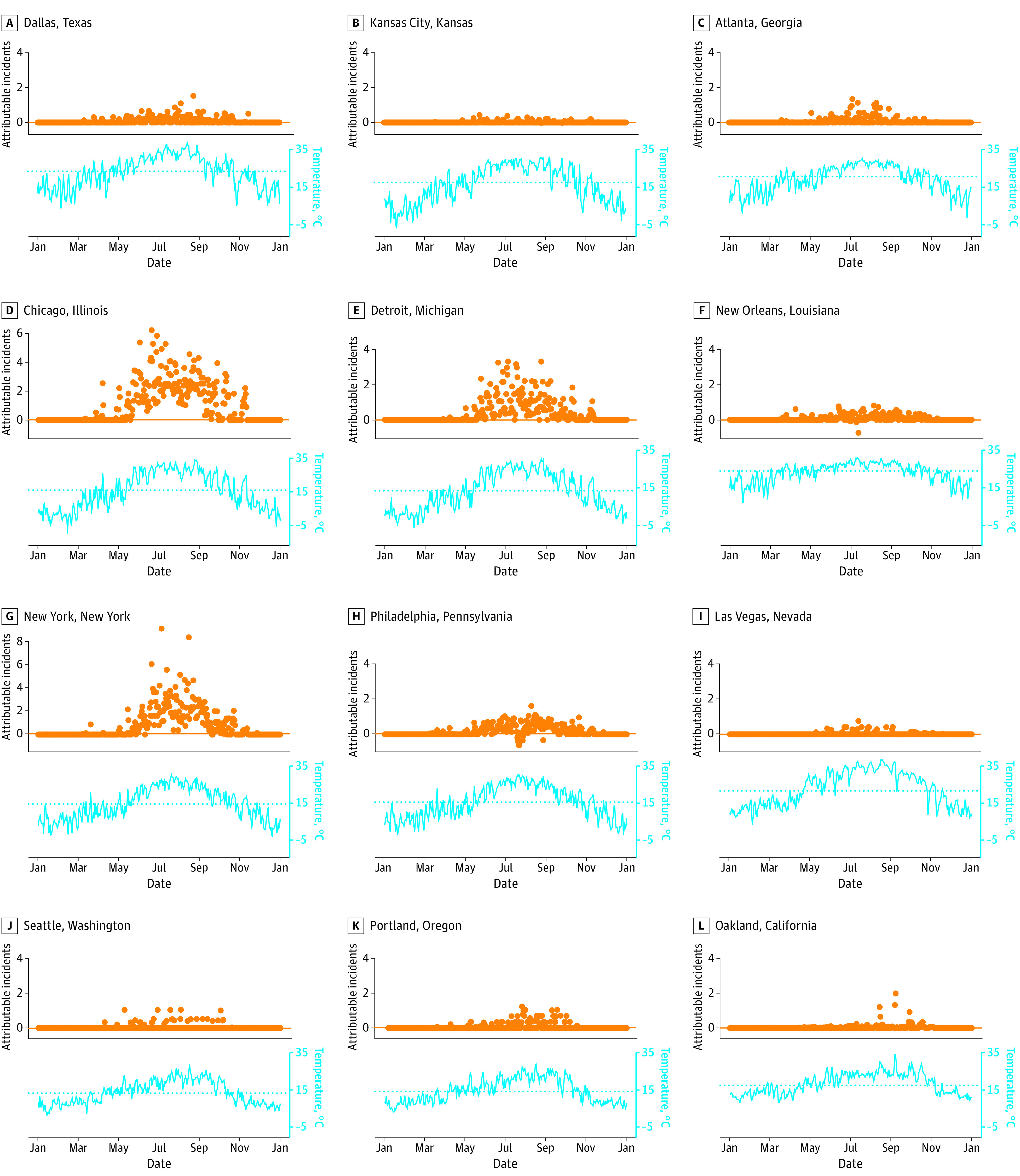
Daily Shootings Attributable to Hot Temperatures in 12 Cities in 2020 These graphs display the count of daily shootings (orange dots) by city in 2020 that can be attributed to hot temperatures (higher than the median for the city), beyond what would be expected in the city for the season and day of the week. Cyan lines show the daily temperatures in each city over the year, and the horizontal dotted line shows the median temperature. These results are based on a model with 0 lag days.

Although the overall shape of the curve representing the association between temperature and shootings at the city level was similar to the pooled analysis, the significance and magnitude of the association, as well as the maximum incident temperature, varied between cities (Figure 3 and eTable 2, eFigure 6, and eFigure 7 in Supplement 1). For example, both Seattle (RR, 1.39; 95% CI, 1.08-1.79) and Las Vegas (RR, 1.14; 95% CI, 0.90-1.46) experience the highest elevated risk of shootings around the 96th temperature percentile, but this corresponds to 84 °F (29 °C) in Seattle and 104 °F (40 °C) in Las Vegas. The number of attributable shootings also differs between cities on the basis of the total number of shootings experienced in a city, and increased risk of a shooting is not confined to just summer months (Figure 4).

In the sensitivity analyses, the 1-day lag model had a higher overall risk of shootings attributable to hot days than observed in any of the other lag day models (AR, 12.22%; 95% CI, 10.45%-13.18%), suggesting that same-day heat may be associated with increased risk of shootings on the following day as well. Although the 0-day and 1-day lag models observed a potential increase in risk at higher temperatures, the 2-day to 7-day lag models showed a potential decrease in risk at higher temperatures (eFigure 5 in Supplement 1). Results of the Cochran *Q* test were not significant (α = .05) with the 1-day, 2-day, 3-day, or 7-day lag models, indicating residual heterogeneity in the daily temperature and shooting association across cities was low (eTable 1 in Supplement 1). Results of the sensitivity analyses restricting the 2020 data and adjusting the pandemic period did not substantively differ from the primary analysis (eFigure 6 in Supplement 1).

## Discussion

In this cross-sectional, time-series study, we found a positive association between daily temperature and shootings that was apparent across the 100 cities with the highest numbers of shootings in the US, with nearly 8000 shootings attributable to above-average daily temperatures. A larger proportion of risk was attributable to moderate heat days than extreme heat days. Heat-attributable shootings were most common in the summer months but were also found in nonsummer months with above-average temperatures. The magnitude of our finding that 6.85% of shootings were attributable to above-average daily temperature indicates that daily temperature may represent a large contributor to firearm violence. Our estimate is also in line with overall global all-cause mortality attributable to nonoptimal temperatures found by Gasparrini et al (7.3%),^24^ indicating that gun violence is a health crisis associated with nonoptimal heat. Although our findings are consistent with prior work identifying an increased risk of firearm violence on warmer days in certain US cities,^11^ the current study is the first, to our knowledge, to identify these associations, and regional variation, over a large number of US cities.

Our findings are distinct from seasonal trends (Figure 4). The daily attributable incident shooting plots suggest that unseasonably warm days can still be associated with increased risk of shootings beyond what would be expected for the season, even when the absolute temperatures are not extreme on an annual basis, and is not constrained to summer months alone. This finding strengthens the support for theories that propose ambient heat as a cause of community violence (eg, temperature-aggression hypothesis and routine activities theory), although our work did not specifically assess these theories. Moreover, it suggests that climate change, which may elevate daily temperatures above normal ranges,^29^ may contribute to increased firearm violence over time.^30^ Specifically, these changes could (1) extend the high-risk season farther into the spring and fall and (2) increase the number of hot summer days when risk is highest.

We found that the association between daily temperature and incident shootings was approximately monotonic over the range of daily temperatures in the pooled analysis and in individual city-level analyses, with no observed specific temperature threshold with a shift or slope change in risk. Accordingly, a larger proportion of shootings was attributable to moderate (50th-89th percentile) temperatures than to extreme (90th-99th percentile) daily temperatures, although the hottest days tend to have the highest incidence rate ratios. The overall monotonic relationship observed suggests a potential dose-response association between heat and firearm violence, which may have additional implications for prevention.

However, these efforts must not ignore the structural context in which communities of color, particularly Black and Indigenous communities, experience disproportionate exposure to urban heat and other effects of climate change.^31^ Historical redlining and disinvestment of resources from redlined communities has situated people of color in neighborhoods with less green space and more asphalt, increasing ambient heat relative to other neighborhoods, and they may be at higher risk of firearm-related violence resulting from elevated temperatures compared with their counterparts in the same city in neighborhoods with more heat-mitigating features.^32^ Future work should examine the intracity dimensions of the heat-violence association through this lens, with an eye toward larger-scale efforts to reduce racial disparities in exposure to both firearm violence and urban heat.

We found regional variation suggesting that above-average daily temperatures may play a different role in firearm violence depending on the climate for a given region, and locales with the greatest variability in temperature may have the greatest associations between heat and shootings. The Northeast and Midwest, where daily median temperatures are the lowest but with high variability, experience the largest fraction of shootings attributable to above-average temperatures. City-specific curves similarly have notable variation in the strength of association and the temperature at which the highest risk of shootings occur, a finding in line with previous work.^33^ These differences may be explained by the era at the time housing was built in a given region or city, as well as policy measures.^15^ Other regional weather patterns like rainfall may also play a role, and future research should examine which climate or built environment factors, if any, explain the variation in these patterns to identify potential prevention opportunities. Our findings also shed light on previous studies^11,12,13^ of heat and violence that have had more limited geographic scope and have conflicting findings around whether temperature is related to increased rates of violence. An increase in warmer temperatures and more frequent extreme heat events due to climate change may create environments with higher risk of firearm violence in the future. Cities and regional governing bodies should consider implementing climate adaptation strategies, either as primary or secondary prevention. Additionally, heat adaptation strategies at the community level that have been effective at reducing urban heat islands (eg, greening efforts or shades) provide general relief from heat may provide additional benefit at reducing shootings.^15^ Some of these interventions, such as increasing green space, have been associated with reductions in firearm violence, potentially through mechanisms unrelated to daily temperature.^34^ Therefore, investments in firearm violence and heat mitigation could potentially produce synergistic effects.

### Limitations

Our study should be considered in light of its limitations. The GVA data have been criticized as not being a fully comprehensive data source on exact numbers of shootings,^35^ but our reliance on shooting is likely more conservative and reliable than specific number of individuals injured or killed. Although we used a sample of 100 cities with high shooting burdens across the US, we were underpowered to fully assess regional variation. We selected the 100 cities with highest numbers of shootings to support our analysis, but it is possible that factors associated with firearm violence in cities and towns with very few shootings may differ substantively from cities with higher numbers. This is an area worth future study. We selected the median temperature within each city as our reference temperature for classifying hot days, but a different choice of comparison group may produce different results. Future studies could examine change in temperature, or some other comparison to examine the shootings attributable to heat below median temperatures.

## Conclusions

Given the increasing rates of firearm violence in the US, the findings of this cross-sectional, time-series study underscore the importance of exploring heat mitigation strategies as tools to reduce shootings, aiming to reduce the impact of daily heat by deploying more resources on warmer days, not just on the hottest (extreme heat) days. Because climate change may shift weather patterns, changes may lead to increase both an increase in overall temperature and frequency of extreme temperatures. Future work should examine this, as well as the roles of racial and economic inequality to address historical inequities in built environment and housing discrimination that create continued disproportionality in shooting incidents among communities of color.

## Notes

Supplement 1. **eAppendix.** Supplemental Methods
eReferences
**eFigure 1.** Graph of Daily Firearm Incidents Across the 100 Cities for the Study Period from 2015-2020 Showing Seasonal Trends **eFigure 2.** Pooled Lagged Relationship Between Firearm Incidents and Temperature Over 7 Days Across 100 US Cities **eFigure 3.** Sensitivity Analysis Changing the Placement and Number of Knots Within the Maximum Daily Temperature and Firearm Incident Relationship, Specified With a 0-Day Lag and Pooled Across 100 US Cities **eFigure 4.** Sensitivity Analysis Changing the Placement and Number of Knots Within the Maximum Daily Temperature and Firearm Incident Relationship, Specified With a 2-Day Lag and Pooled Across 100 US Cities **eFigure 5.** Sensitivity Analysis Assessing the Pooled Firearm Incident and Temperature Association Using Different Numbers of Lag Days, Across 100 US Cities **eFigure 6.** Results of the Overall Heat and Firearm Incident Association in the Sensitivity Analyses Accounting for the 2020 Pandemic Period **eTable 1.** Sensitivity Analyses Assessing the Excess Risk of Firearm Incidents on Hot Days Using Different Numbers of Lag Days **eTable 2.** Analysis City Characteristics and Attributable Heat Estimates From the Zero-Lag Model **eFigure 7.** 100 City-Specific Result Graphs

Supplement 2.
Data Sharing Statement
