# Supplementary material for: Analysis of Daily Ambient Temperature and Firearm Violence in 100 US Cities
Source: JAMA Netw Open. 2022 Dec 16;5(12):e2247207. doi: 10.1001/jamanetworkopen.2022.47207 (PMC9856408; doi:10.1001/jamanetworkopen.2022.47207)
Supplement: Supplement 1. — eAppendix. Supplemental Methods eReferences eFigure 1. Graph of Daily Firearm Incidents Across the 100 Cities for the Study Period from 2015-2020 Showing Seasonal Trends eFigure 2. Pooled Lagged Relationship Between Firearm Incidents and Temperature Over 7 Days Across 100 US Cities eFigure 3. Sensitivity Analysis Changing the Placement and Number of Knots Within the Maximum Daily Temperature and Firearm Incident Relationship, Specified With a 0-Day Lag and Pooled Across 100 US Cities eFigure 4. Sensitivity Analysis Changing the Placement and Number of Knots Within the Maximum Daily Temperature and Firearm Incident Relationship, Specified With a 2-Day Lag and Pooled Across 100 US Cities eFigure 5. Sensitivity Analysis Assessing the Pooled Firearm Incident and Temperature Association Using Different Numbers of Lag Days, Across 100 US Cities eFigure 6. Results of the Overall Heat and Firearm Incident Association in the Sensitivity Analyses Accounting for the 2020 Pandemic Period eTable 1. Sensitivity Analyses Assessing the Excess Risk of Firearm Incidents on Hot Days Using Different Numbers of Lag Days eTable 2. Analysis City Characteristics and Attributable Heat Estimates From the Zero-Lag Model eFigure 7. 100 City-Specific Result Graphs [file jamanetwopen-e2247207-s001.pdf]

Supplemental Online Content

Lyons VH, Gause EL, Spangler KR, Wellenius GA, Jay J. Analysis of daily ambient temperature and firearm violence in 100 US cities. *JAMA Netw Open*. 2022;5(12):e2247207. doi:10.1001/jamanetworkopen.2022.47207

eAppendix. Supplemental Methods
eReferences
eFigure 1. Graph of Daily Firearm Incidents Across the 100 Cities for the Study Period from 2015-2020 Showing Seasonal Trends
eFigure 2. Pooled Lagged Relationship Between Firearm Incidents and Temperature Over 7 Days Across 100 US Cities
eFigure 3. Sensitivity Analysis Changing the Placement and Number of Knots Within the Maximum Daily Temperature and Firearm Incident Relationship, Specified With a 0-Day Lag and Pooled Across 100 US Cities
eFigure 4. Sensitivity Analysis Changing the Placement and Number of Knots Within the Maximum Daily Temperature and Firearm Incident Relationship, Specified With a 2-Day Lag and Pooled Across 100 US Cities
eFigure 5. Sensitivity Analysis Assessing the Pooled Firearm Incident and Temperature Association Using Different Numbers of Lag Days, Across 100 US Cities
eFigure 6. Results of the Overall Heat and Firearm Incident Association in the Sensitivity Analyses Accounting for the 2020 Pandemic Period
eTable 1. Sensitivity Analyses Assessing the Excess Risk of Firearm Incidents on Hot Days Using Different Numbers of Lag Days
eTable 2. Analysis City Characteristics and Attributable Heat Estimates From the Zero-Lag Model
eFigure 7. 100 City-Specific Result Graphs

This supplemental material has been provided by the authors to give readers additional information about their work.

© 2022 Lyons VH et al. *JAMA Network Open*.

eAppendix. Supplemental Methods

Notes on analysis, data, and computation:
All analyses were performed in R Version 4.0.5[1] using the following packages: dlnm,[2] mvmeta,[3] tsModel,[4] and splines.[1]

The code used in the primary analysis can be found on GitHub: https://github.com/Epi-Emma/2022_temperature_and_firearm_violence.git

This analysis benefited from the R code examples written by Antonio Gasparrini and made available on his personal website (http://www.ag-myresearch.com/):

- Example of a DLNM analysis across multiple locations: https://github.com/gasparrini/2015_gasparrini_Lancet_Rcodedata
- Investigating the lagged relationship: https://github.com/gasparrini/2016_gasparrini_AJE_Rcodedata
- Attributable risk calculations and plots: https://github.com/gasparrini/2014_gasparrini_BMCmrm_Rcodedata

US Census Bureau administrative "places" shapefiles were used to define the geographic boundaries for each of the 100 analysis cities.[5] We used population estimates derived from the Global Human Settlements Population (GHS-POP) 2015 dataset for the population weighting of the city-specific maximum daily temperatures from the North American Land Data Assimilation System Phase 2 (NLDAS-2).[6-8]

eReferences

1. R Core Team (2021). R: A language and environment for statistical computing. R Foundation for Statistical Computing, Vienna, Austria. URL https://www.R-project.org/.

2. Gasparrini A. Distributed lag linear and non-linear models in R: the package dlnm. Journal of Statistical Software. 2011; 43(8):1-20. https://doi.org/10.18637/jss.v043.i08

3. Gasparrini A., Armstrong, B., Kenward M. G. (2012). Multivariate meta-analysis for non-linear and other multi-parameter associations. Statistics in Medicine. 31(29):3821-3839.

4. Roger D. Peng and with contributions from Aidan McDermott (2022). tsModel: Time Series Modeling for Air Pollution and Health. R package version 0.6-1. https://CRAN.R-project.org/package=tsModel

5. US Census Bureau. "Cartographic Boundary Files - Shapefile." Accessed November 11, 2021. https://www.census.gov/geographies/mapping-files/time-series/geo/carto-boundary-file.2010.html

© 2022 Lyons VH et al. *JAMA Network Open*.

6. Schiavina M, Freire S, MacManus K. GHS-POP R2019A - GHS population grid multitemporal (1975-1990-2000-2015) [Internet]. Jt. Res. Centre, Eur. Comm. 2019;Available from: https://data.europa.eu/euodp/en/data/dataset/0c6b9751-a71f-4062-830b-43c9f432370f

7. Xia, Y., et al., NCEP/EMC (2009), NLDAS Primary Forcing Data L4 Hourly 0.125 x 0.125 degree V002, Edited by David Mocko, NASA/GSFC/HSL, Greenbelt, Maryland, USA, Goddard Earth Sciences Data and Information Services Center (GES DISC), Accessed: March 1, 2020, DOI: 10.5067/6J5LHHOHZHN4

8. Xia, Y., K. Mitchell, M. Ek, J. Sheffield, B. Cosgrove, E. Wood, L. Luo, C. Alonge, H. Wei, J. Meng, B. Livneh, D. Lettenmaier, V. Koren, Q. Duan, K. Mo, Y. Fan, and D. Mocko (2012). Continental-scale water and energy flux analysis and validation for the North American Land Data Assimilation System project phase 2 (NLDAS-2): 1. Intercomparison and application of model products. J. Geophys. Res., 117, D03109, doi:10.1029/2011JD016048

© 2022 Lyons VH et al. *JAMA Network Open*.

eFigure 1: Graph of Daily Firearm Incidents Across the 100 Cities for the Study Period from 2015-2020 Showing Seasonal Trends

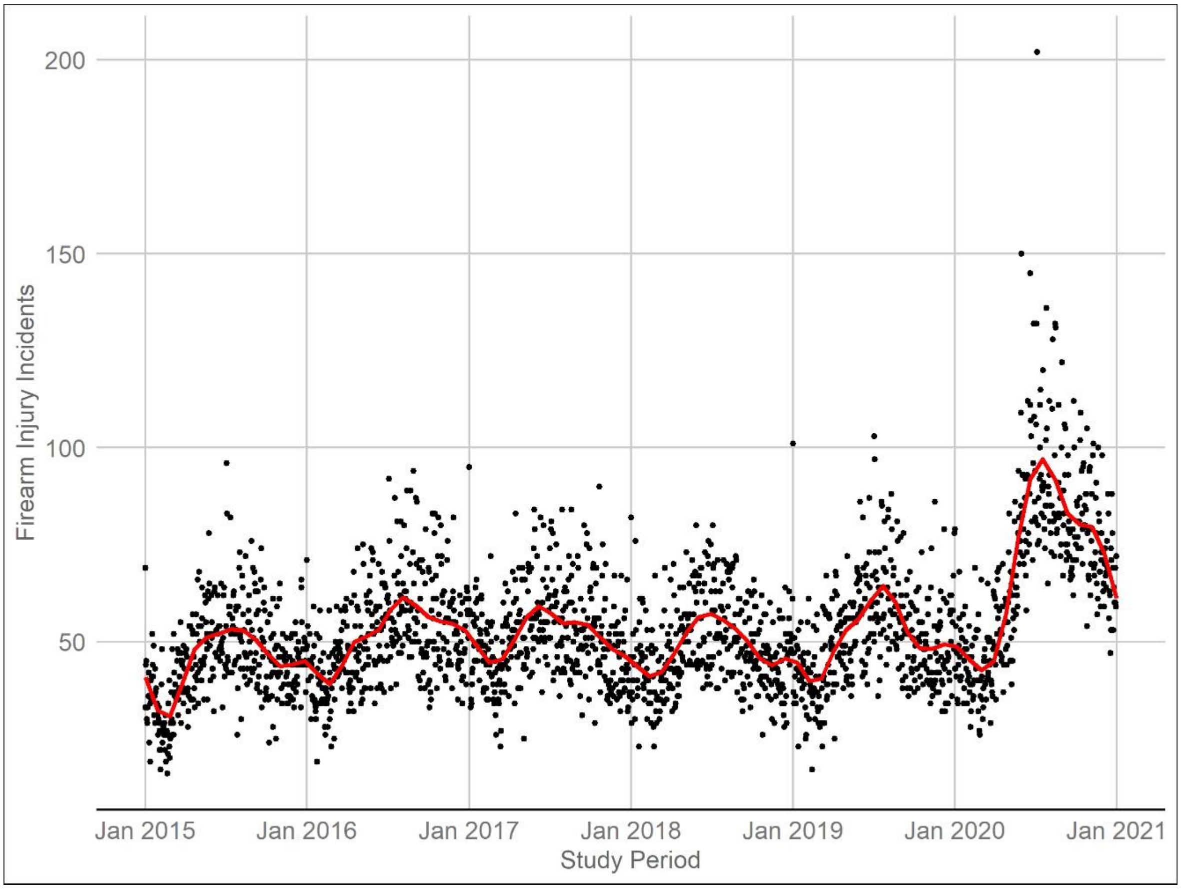

Note: This figure plots the daily count of firearm incidents among the 100 analysis cities throughout the study period from 2015 through 2020. Daily firearm incident counts range from 16 to 202 per day (the high outlier of 202 firearm incidents occurred on July 5th, 2020). The red line is represents our method for controlling for seasonality: cubic splines with 7 knots per study year for a total of 42 knots). In the primary analyses, adjustment for seasonal trends was city-specific before results were pooled across cities, so this graph is just an example of what the seasonal trends would look like using all cities' data.

© 2022 Lyons VH et al. *JAMA Network Open*.

eFigure 2: Pooled Lagged Relationship Between Firearm Incidents and Temperature Over 7 Days Across 100 US Cities

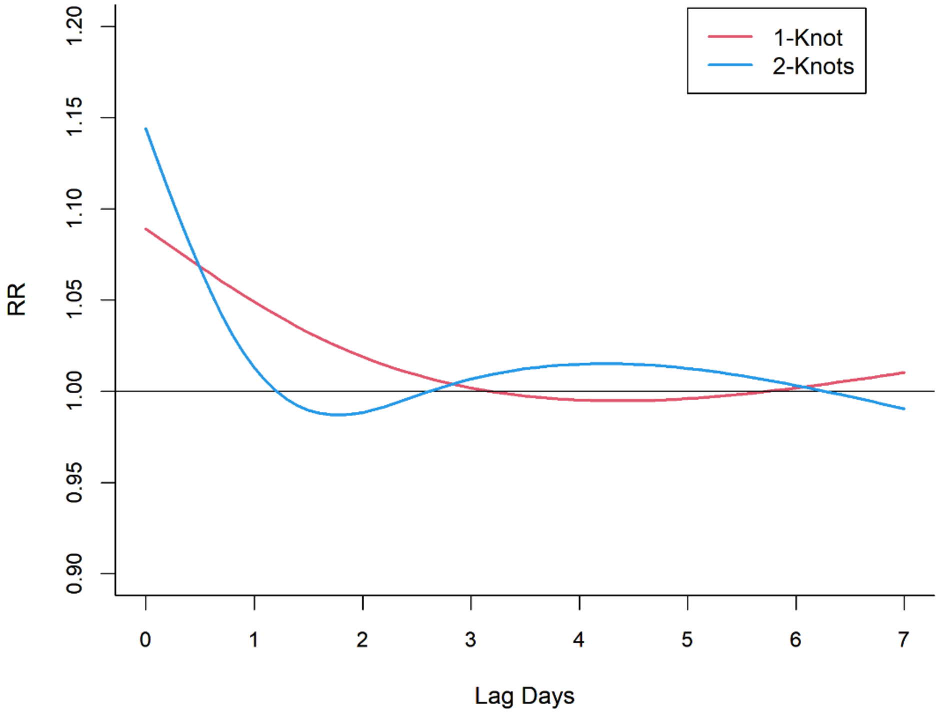

NOTE: This graph displays the overall pooled lagged function comparing the risk of firearm incidents at the 95th temperature percentile compared to the median temperature from a DLNM model fit with up to seven days of lag across 100 cities. The DLNM models were fit with knots at the 10th, 75th, and 90th percentiles for the exposure~outcome association. A sensitivity analysis was conducted using both one and two inner knots within the lag~outcome association to allow greater flexibility in the lagged relationship. The influence of heat on firearm incidents was strongest on the day of, which prompted the authors to choose a zero-lag model for the primary analysis.

© 2022 Lyons VH et al. *JAMA Network Open*.

eFigure 3: Sensitivity Analysis Changing the Placement and Number of Knots Within the Maximum Daily Temperature and Firearm Incident Relationship, Specified With a 0-Day Lag and Pooled Across 100 US Cities

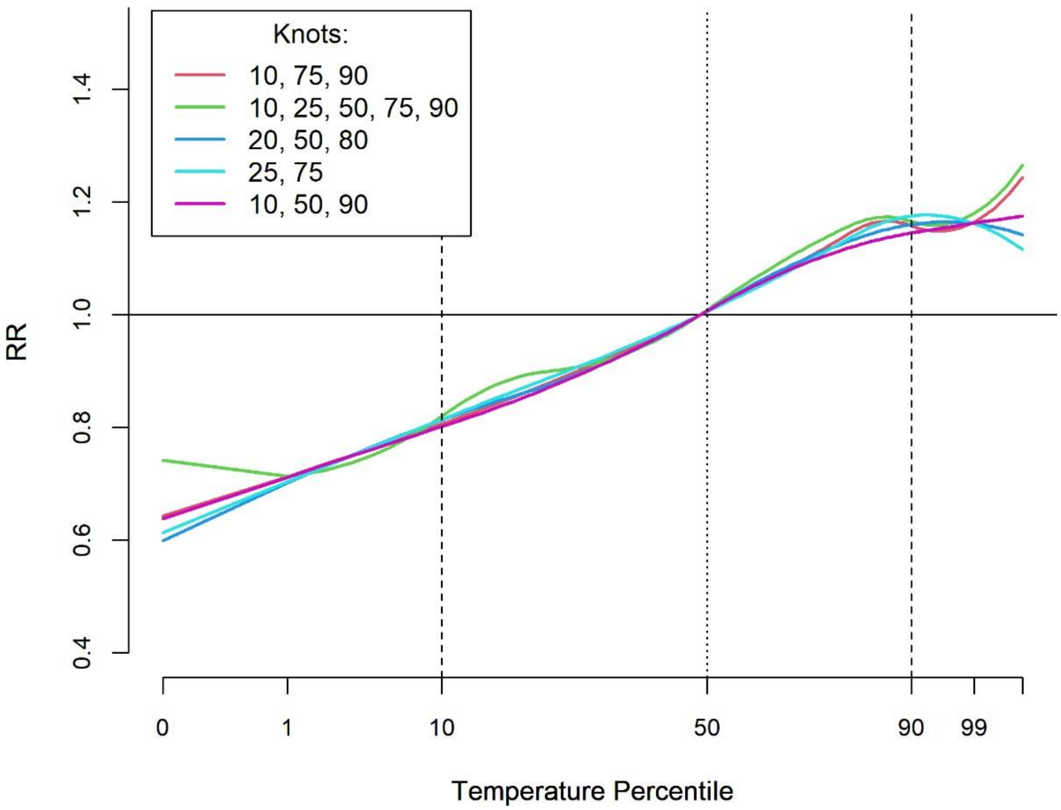

Note: This graph depicts the overall pooled association between daily maximum temperature and firearm incidents across 100 cities, with different combinations of the number and placement of knots specified for the exposure~outcome association to assess whether the relationship was robust to model specification. The DLNM models were fit with a 0-day lag period.

© 2022 Lyons VH et al. *JAMA Network Open*.

eFigure 4: Sensitivity Analysis Changing the Placement and Number of Knots Within the Maximum Daily Temperature and Firearm Incident Relationship, Specified With a 2-Day Lag and Pooled Across 100 US Cities

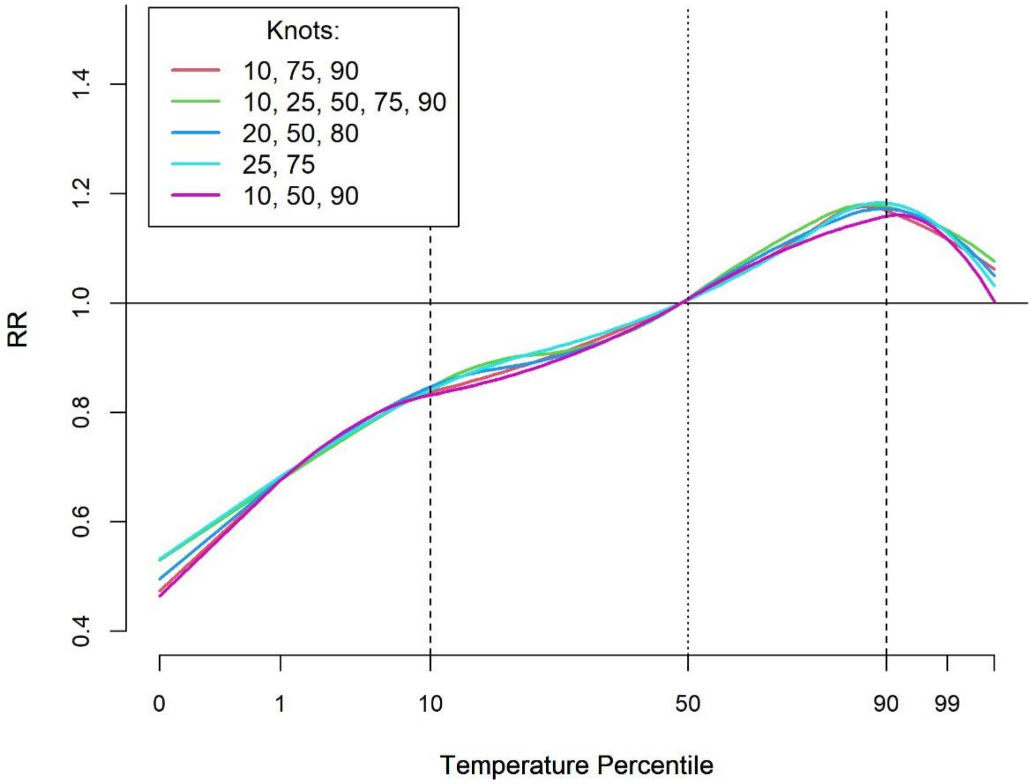

Note: This graph depicts the overall pooled association between daily maximum temperature and firearm incidents across 100 cities, with different combinations of the number and placement of knots specified for the exposure~outcome association to assess whether the relationship was robust to model specification. The DLNM models were fit with a 2-day lag period.

© 2022 Lyons VH et al. *JAMA Network Open*.

eFigure 5: Sensitivity Analysis Assessing the Pooled Firearm Incident and Temperature Association Using Different Numbers of Lag Days, Across 100 US Cities

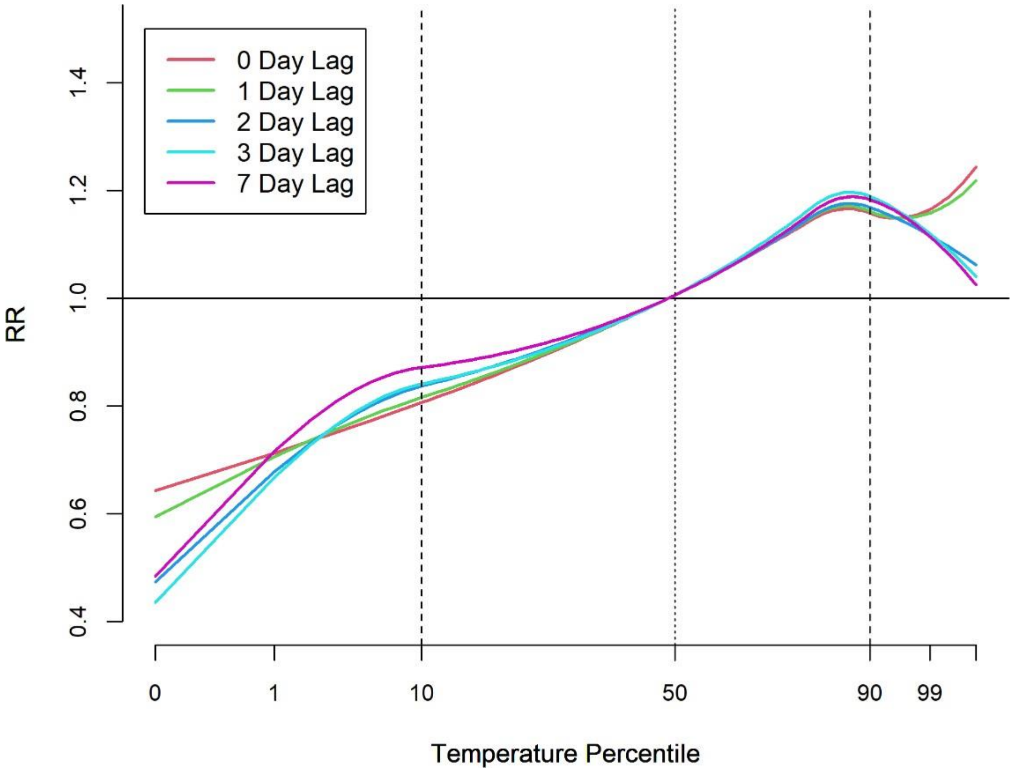

Note: This graph depicts the overall pooled association between daily maximum temperature and firearm incidents across 100 cities, using varying lag durations to assess how the relationship changes with the inclusion of increasing lagged days. The DLNM models were fit with knots at the 10th, 75th, and 90th percentiles. The relative risks depicted here represent the cumulative risk over the specific lag duration at each temperature percentile, pooled across the 100 cities.

© 2022 Lyons VH et al. *JAMA Network Open*.

eFigure 6: Results of the Overall Heat and Firearm Incident Association in the Sensitivity Analyses Accounting for the 2020 Pandemic Period

A) Including an indicator for the pandemic period (March 1, 2020 – December 31, 2020)

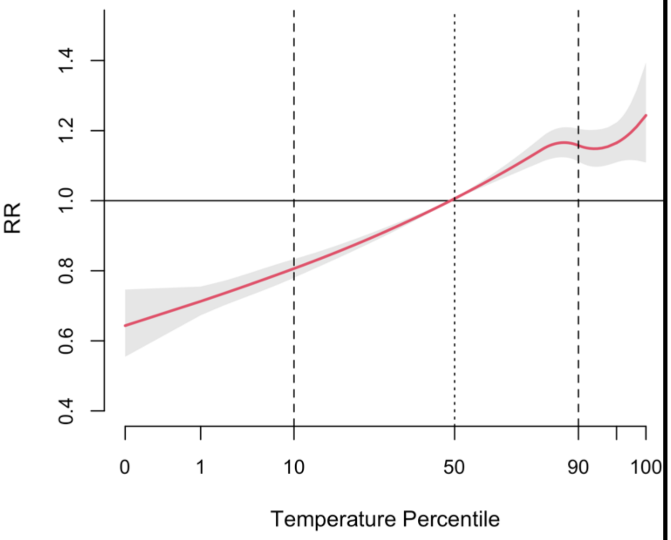

	Attributable Fraction (95% CI)
**Total Heat**	6.85 (6.03, 7.44)
Moderate Heat	5.00 (4.45, 5.46)
Extreme Heat	1.86 (1.55, 2.04)

B) Restricting data years to remove 2020 from analysis

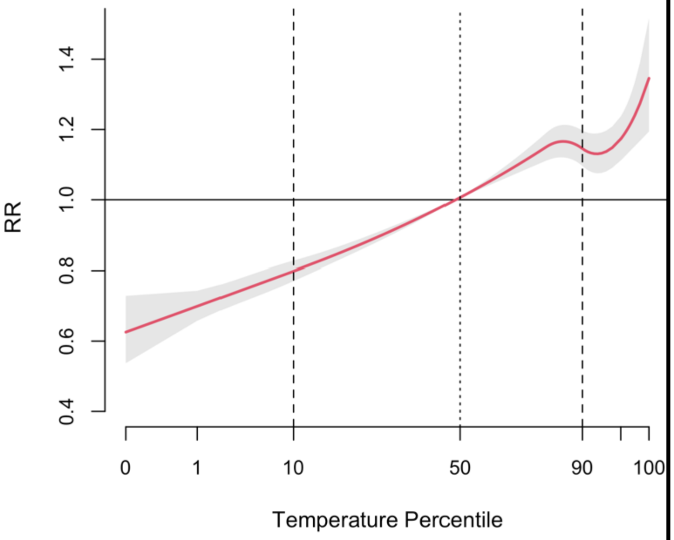

	Attributable Fraction (95% CI)
**Total Heat**	6.30 (5.40, 6.95)
Moderate Heat	4.70 (4.01, 5.18)
Extreme Heat	1.61 (1.31, 1.83)

Note: These graphs depict the overall pooled association between daily maximum temperature and firearm incidents across 100 cities using two different approaches to account for the influence of the pandemic period. Both analyses are modeled similarly to the primary analysis with knots at the 10th, 75th, and 90th percentiles and including zero lag days.

© 2022 Lyons VH et al. *JAMA Network Open*.

eTable 1: Sensitivity Analyses Assessing the Excess Risk of Firearm Incidents on Hot Days Using Different Numbers of Lag Days

	0 Day Lag	1 Day Lag	2 Day Lag	3 Day Lag	7 Day Lag
**Attributable Risk Fraction**					
**All heat**	6.85 (6.09, 7.46)	12.22 (10.45, 13.18)	6.73 (5.75, 7.46)	7.29 (6.23, 8.05)	7.17 (6.05, 7.86)
**Moderate heat**	5.00 (4.44, 5.43)	9.10 (7.78, 9.80)	5.02 (4.23, 5.55)	5.40 (4.63, 5.96)	5.40 (4.63, 5.96)
**Extreme Heat**	1.86 (1.58, 2.05)	3.14 (2.47, 3.41)	1.72 (1.39, 1.93)	1.90 (1.55, 2.12)	1.78 (1.42, 1.96)
**Attributable Risk Number**					
**All heat**	7973 (7092, 8688)	14239 (12178, 15355)	7842 (6697, 8691)	8494 (7254, 9378)	8350 (7053, 9162)
**Moderate heat**	5820 (5173, 6329)	10607 (9066, 11416)	5848 (4929, 6466)	6287 (5397, 6942)	6288 (5392, 6941)
**Extreme Heat**	2164 (1839, 2388)	3654 (2875, 3977)	2005 (1624, 2243)	2219 (1800, 2467)	2073 (1654, 2279)
**I^2 Statistic**	11.7%	8.5%	4.3%	4.3%	5.6%
**Cochran's Q Test**	p=0.02	p=0.08	p=0.17	p=0.24	p=0.18

Note: The model with the 7-day lag included two internal knots to model the lagged temperature and firearm relationship. The 2- and 3-day lag models included a single internal knot in the lagged association. The 1-day lag model did not include any internal knots. The 0-day lag model necessarily included no lag at all.

© 2022 Lyons VH et al. *JAMA Network Open*.

eTable 2: Analysis City Characteristics and Attributable Heat Estimates From the Zero-Lag Model

City	Climate Region	Total Firearm Incidents	Median Temp (F)	Max Incident Temp Perc.	Max Incident Temp (F)	Heat Attributable Incidents (%)	Heat Attributable Incidents (95% CI)
**Akron, OH**	Midwest	551	62.7	79	80.0	6.53	(0.98, 11.77)
**Albuquerque, NM**	Southwest	608	71.0	96	95.5	5.12	(-0.98, 10.47)
**Atlanta, GA**	Southeast	1172	75.2	96	92.3	5.39	(0.32, 9.84)
**Augusta, GA**	Southeast	373	78.4	76	89.5	1.60	(-4.72, 6.93)
**Austin, TX**	Great Plains	398	83.6	77	94.6	1.66	(-4.46, 7.4)
**Bakersfield, CA**	Southwest	367	82.7	80	103.4	2.62	(-5.85, 9.09)
**Baltimore, MD**	Northeast	4659	65.8	78	82.3	6.15	(2.03, 9.77)
**Baton Rouge, LA**	Southeast	874	82.1	66	86.5	-0.36	(-4.91, 3.46)
**Birmingham, AL**	Southeast	1131	76.1	74	86.1	-0.35	(-5.91, 4.4)
**Boston, MA**	Northeast	907	59.5	91	84.5	12.51	(5.76, 18.45)
**Bridgeport, CT**	Northeast	523	61.1	92	84.9	12.47	(5.37, 17.89)
**Buffalo, NY**	Northeast	848	58.3	93	84.8	12.88	(5.84, 18.78)
**Charlotte, NC**	Southeast	1249	73.8	77	87.3	4.94	(-0.51, 9.59)
**Chattanooga, TN**	Southeast	727	73.7	81	86.7	6.24	(0.26, 11.24)
**Chicago, IL**	Midwest	16136	61.0	86	83.6	10.69	(7.51, 13.83)
**Cincinnati, OH**	Midwest	1185	67.0	96	90.3	5.37	(-0.56, 10.57)
**Cleveland, OH**	Midwest	1992	61.8	81	80.6	9.94	(4.73, 14.61)
**Colorado Springs, CO**	Southwest	498	61.3	95	86.6	6.79	(1.23, 11.4)
**Columbus, GA**	Southeast	527	79.0	74	88.2	0.26	(-5.24, 5.01)
**Columbus, OH**	Midwest	1648	65.4	83	84.5	6.32	(1.11, 10.74)
**Dallas, TX**	Great Plains	1352	80.8	82	96.5	5.33	(-0.59, 10.65)
**Dayton, OH**	Midwest	653	65.9	96	90.3	5.13	(-0.75, 10.39)
**Denver, CO**	Southwest	700	64.7	87	88.4	7.07	(0.73, 12.42)
**Detroit, MI**	Midwest	2877	60.5	94	87.9	11.60	(6.62, 15.59)
**Durham, NC**	Southeast	598	73.5	87	90.2	5.65	(0.11, 10.53)
**Flint, MI**	Midwest	463	58.4	93	85.8	8.72	(1.27, 14.24)
**Fort Wayne, IN**	Midwest	507	63.2	87	84.9	7.14	(0.45, 12.49)
**Fort Worth, TX**	Great Plains	758	80.8	80	95.8	1.72	(-3.83, 6.85)

© 2022 Lyons VH et al. *JAMA Network Open*.

**Fresno, CA**	Southwest	715	80.4	78	100.5	3.64	(-2.66, 9.22)
**Gary, IN**	Midwest	634	62.1	92	87.1	9.92	(3.48, 14.91)
**Grand Rapids, MI**	Midwest	311	58.4	92	84.9	12.42	(4.29, 18.91)
**Greensboro, NC**	Southeast	540	71.9	76	85.4	2.19	(-3.92, 7.55)
**Hampton, VA**	Southeast	456	68.0	79	81.7	4.99	(-1.46, 10.33)
**Hartford, CT**	Northeast	469	60.6	86	81.8	9.45	(2.96, 15.06)
**Houston, TX**	Great Plains	2999	82.5	80	91.2	3.79	(-0.65, 7.64)
**Huntsville, AL**	Southeast	332	74.4	77	86.2	2.96	(-3.75, 8.54)
**Indianapolis, IN**	Midwest	2150	65.6	96	89.1	6.23	(1.19, 10.62)
**Jackson, MS**	Southeast	1049	79.6	80	89.7	1.85	(-2.86, 6.05)
**Jacksonville, FL**	Southeast	1888	81.5	96	92.4	2.51	(-1.98, 6.24)
**Jersey City, NJ**	Northeast	426	62.9	88	84.4	11.23	(5.38, 16.23)
**Kansas City, KS**	Great Plains	326	68.9	85	89.4	7.86	(0.88, 13.43)
**Kansas City, MO**	Midwest	1365	68.8	93	93.0	6.13	(0.78, 10.73)
**Knoxville, TN**	Southeast	361	72.1	80	85.3	4.04	(-1.66, 9.27)
**Las Vegas, NV**	Southwest	497	76.9	96	104.1	4.57	(-2.57, 9.74)
**Lexington, KY**	Southeast	592	67.5	96	89.1	5.97	(0.09, 10.72)
**Little Rock, AR**	Southeast	881	75.6	79	89.2	4.95	(0.13, 9.25)
**Long Beach, CA**	Southwest	406	66.4	67	69.6	-0.10	(-10.84, 8.37)
**Los Angeles, CA**	Southwest	1363	72.3	58	74.2	-1.08	(-7.59, 3.92)
**Louisville, KY**	Southeast	1529	69.1	96	90.7	4.03	(-0.69, 8.71)
**Macon, GA**	Southeast	427	78.9	78	90.4	2.01	(-3.85, 7.18)
**Memphis, TN**	Southeast	2867	75.5	82	90.5	5.51	(1.01, 9.3)
**Miami, FL**	Southeast	388	83.9	28	80.0	-1.94	(-8.85, 3.82)
**Milwaukee, WI**	Midwest	2867	55.8	93	83.5	11.92	(6.79, 16.99)
**Minneapolis, MN**	Midwest	788	57.8	87	83.7	15.92	(9.04, 22.12)
**Mobile, AL**	Southeast	567	80.3	76	87.7	2.37	(-2.65, 7.36)
**Montgomery, AL**	Southeast	658	79.9	75	89.1	0.62	(-5.08, 5.47)
**Nashville, TN**	Southeast	1645	72.7	75	85.4	2.37	(-2.54, 6.78)
**New Haven, CT**	Northeast	392	60.3	95	85.1	11.58	(4.15, 17.6)
**New Orleans, LA**	Southeast	2767	81.7	80	88.9	3.47	(-0.56, 6.92)
**New York, NY**	Northeast	4302	62.8	87	83.8	15.08	(10.85, 19.28)

© 2022 Lyons VH et al. *JAMA Network Open*.

**Newark, NJ**	Northeast	896	63.0	85	83.3	9.51	(4.25, 14.42)
**Newport News, VA**	Southeast	514	69.4	81	83.9	5.45	(-0.89, 10.71)
**Norfolk, VA**	Southeast	682	69.7	81	83.5	5.26	(-0.51, 10.4)
**North Charleston, SC**	Southeast	383	78.6	96	93.1	2.96	(-3.63, 7.95)
**Oakland, CA**	Southwest	1364	68.1	96	85.6	3.25	(-3.37, 8.94)
**Oklahoma City, OK**	Great Plains	887	75.0	82	93.2	3.95	(-2.26, 9.38)
**Omaha, NE**	Great Plains	721	65.9	82	87.4	8.20	(1.87, 13.45)
**Orlando, FL**	Southeast	384	85.6	76	90.0	0.39	(-4.52, 4.67)
**Paterson, NJ**	Northeast	502	62.2	96	88.2	8.83	(3.52, 13.75)
**Peoria, IL**	Midwest	434	65.3	89	88.6	10.25	(2.77, 16.31)
**Philadelphia, PA**	Northeast	5736	64.7	87	85.2	6.07	(2.1, 9.2)
**Phoenix, AZ**	Southwest	1148	86.8	57	91.3	-1.03	(-8.88, 5)
**Pittsburgh, PA**	Northeast	872	64.5	93	87.1	9.67	(4.31, 14.63)
**Portland, OR**	Northwest	524	61.5	96	85.5	8.71	(1.57, 14.7)
**Portsmouth, VA**	Southeast	414	72.0	81	85.5	4.18	(-1.89, 9.29)
**Raleigh, NC**	Southeast	361	73.7	76	86.6	2.07	(-4.35, 7.31)
**Richmond, VA**	Southeast	1126	71.0	80	86.3	4.33	(-1.59, 9.08)
**Rochester, NY**	Northeast	796	57.9	96	86.4	8.28	(1.5, 14.14)
**Rockford, IL**	Midwest	556	61.0	93	86.8	14.25	(8.12, 19.59)
**San Antonio, TX**	Great Plains	1841	85.3	77	95.5	2.23	(-2.78, 6.44)
**San Diego, CA**	Southwest	385	76.3	54	77.5	-0.96	(-7.1, 4.4)
**San Francisco, CA**	Southwest	421	60.1	80	64.9	2.65	(-6.9, 10.73)
**Savannah, GA**	Southeast	607	80.0	76	87.7	0.82	(-4.58, 5)
**Seattle, WA**	Northwest	440	59.7	96	84.4	12.00	(4.34, 17.75)
**Shreveport, LA**	Southeast	912	81.0	84	93.1	6.07	(1, 10.75)
**South Bend, IN**	Midwest	401	61.5	93	86.6	13.26	(6.35, 19.42)
**Springfield, MA**	Northeast	367	59.8	96	86.3	6.56	(0.2, 12.58)
**St. Louis, MO**	Midwest	2959	69.0	81	87.6	6.68	(1.43, 11.63)
**St. Paul, MN**	Midwest	336	57.7	90	85.0	10.97	(4.73, 16.34)
**Stockton, CA**	Southwest	519	77.1	79	96.6	4.17	(-3.85, 11.2)
**Syracuse, NY**	Northeast	507	58.0	92	83.6	13.29	(7.45, 18.68)
**Toledo, OH**	Midwest	901	61.9	88	85.5	10.07	(3.99, 15.76)

© 2022 Lyons VH et al. *JAMA Network Open*.

**Topeka, KS**	Great Plains	325	69.5	93	94.4	5.67	(-0.67, 10.87)
**Trenton, NJ**	Northeast	527	64.5	96	89.7	8.29	(1.94, 13.46)
**Tucson, AZ**	Southwest	308	83.2	76	95.7	1.83	(-4.66, 7.78)
**Tulsa, OK**	Great Plains	981	74.3	82	91.4	3.92	(-2.36, 8.97)
**Washington, DC**	Northeast	2531	67.4	80	84.5	5.96	(1.13, 9.95)
**Wichita, KS**	Great Plains	587	71.3	92	95.8	6.08	(0.36, 11.16)
**Wilmington, DE**	Northeast	710	65.1	81	83.0	6.19	(0.13, 11.5)
**Winston-Salem, NC**	Southeast	408	71.7	96	91.7	4.92	(-1.47, 10.53)

© 2022 Lyons VH et al. *JAMA Network Open*.

eFigure 7: 100 City- Specific Result Graphs

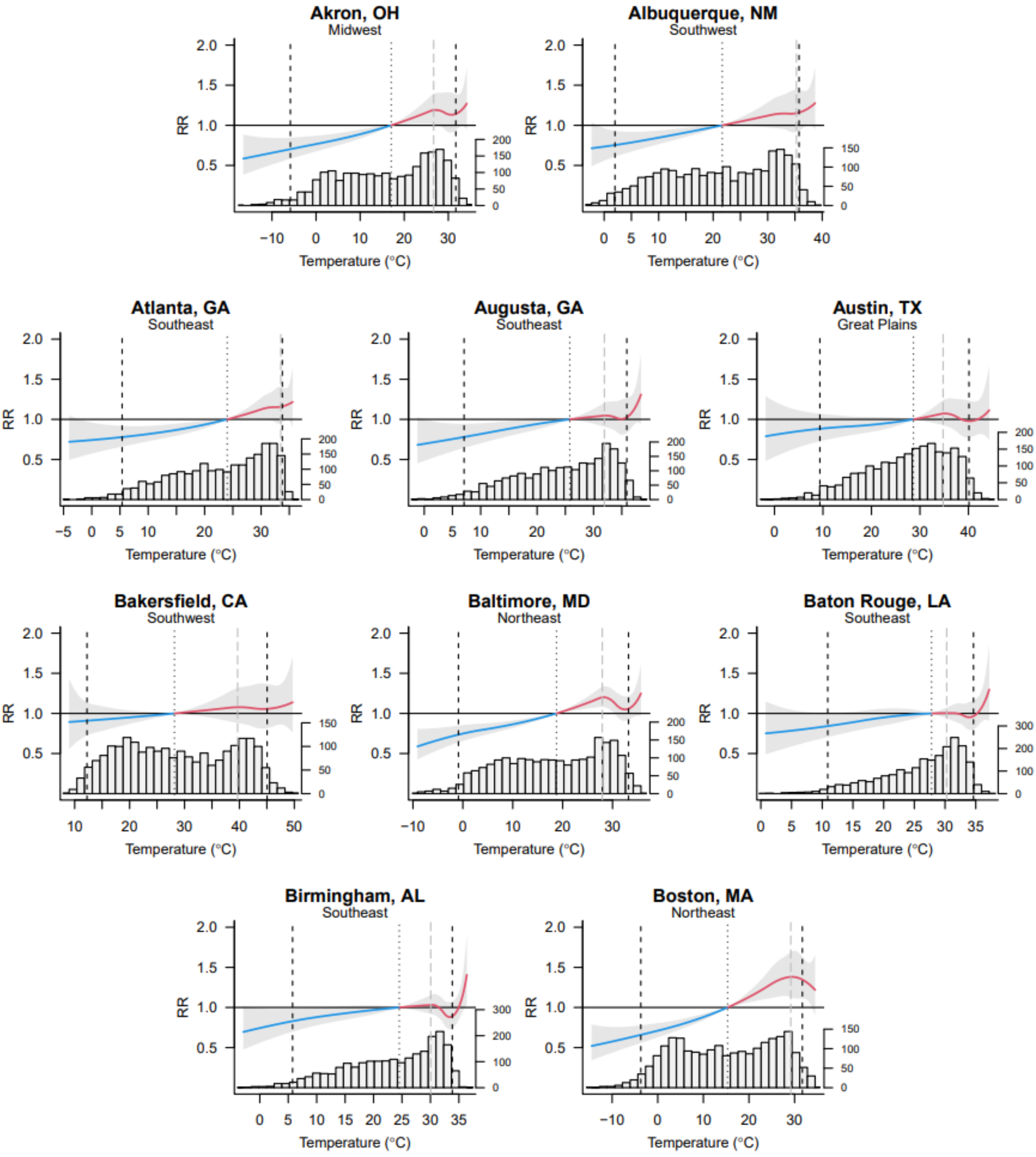

© 2022 Lyons VH et al. *JAMA Network Open*.

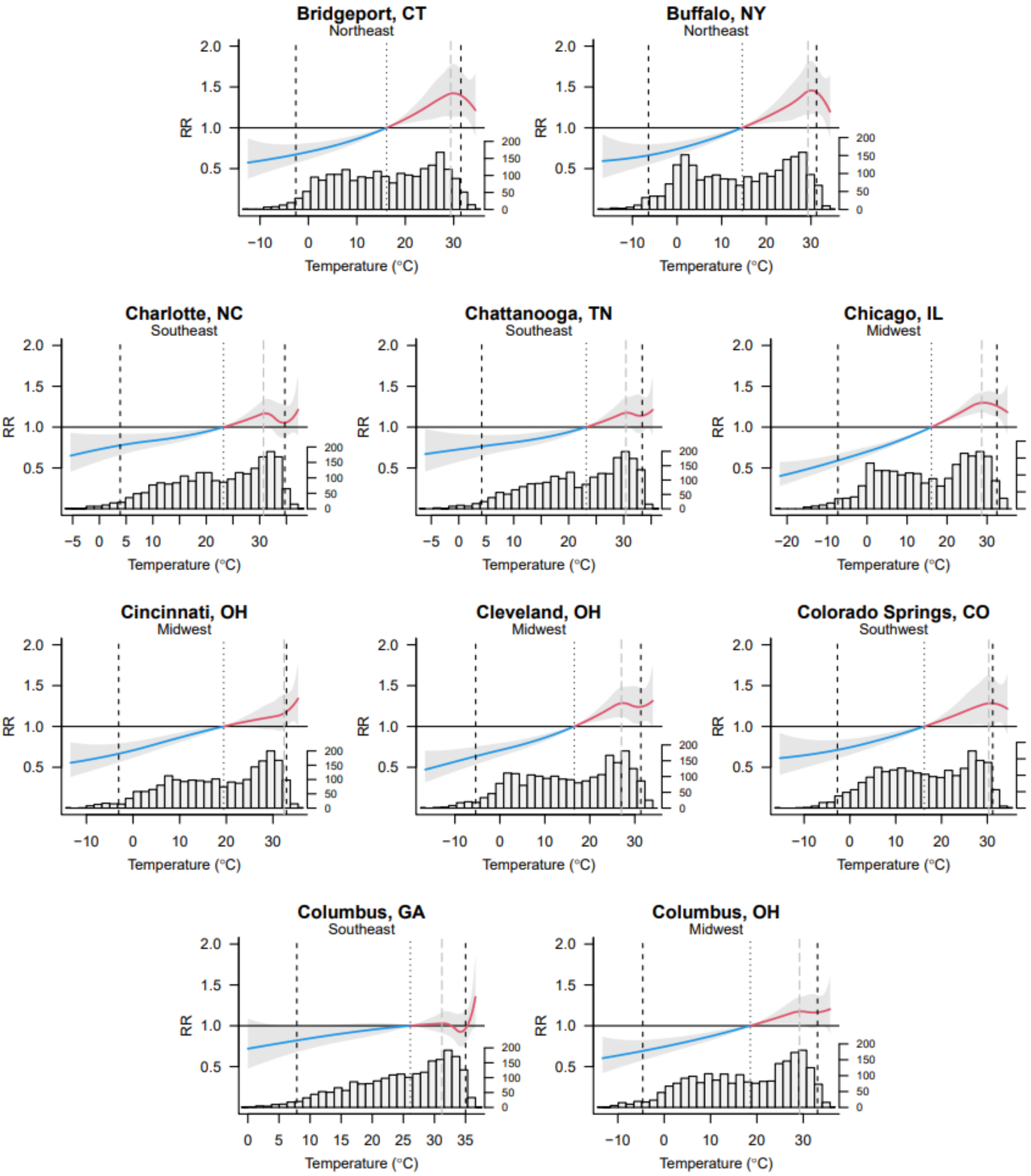

© 2022 Lyons VH et al. *JAMA Network Open*.

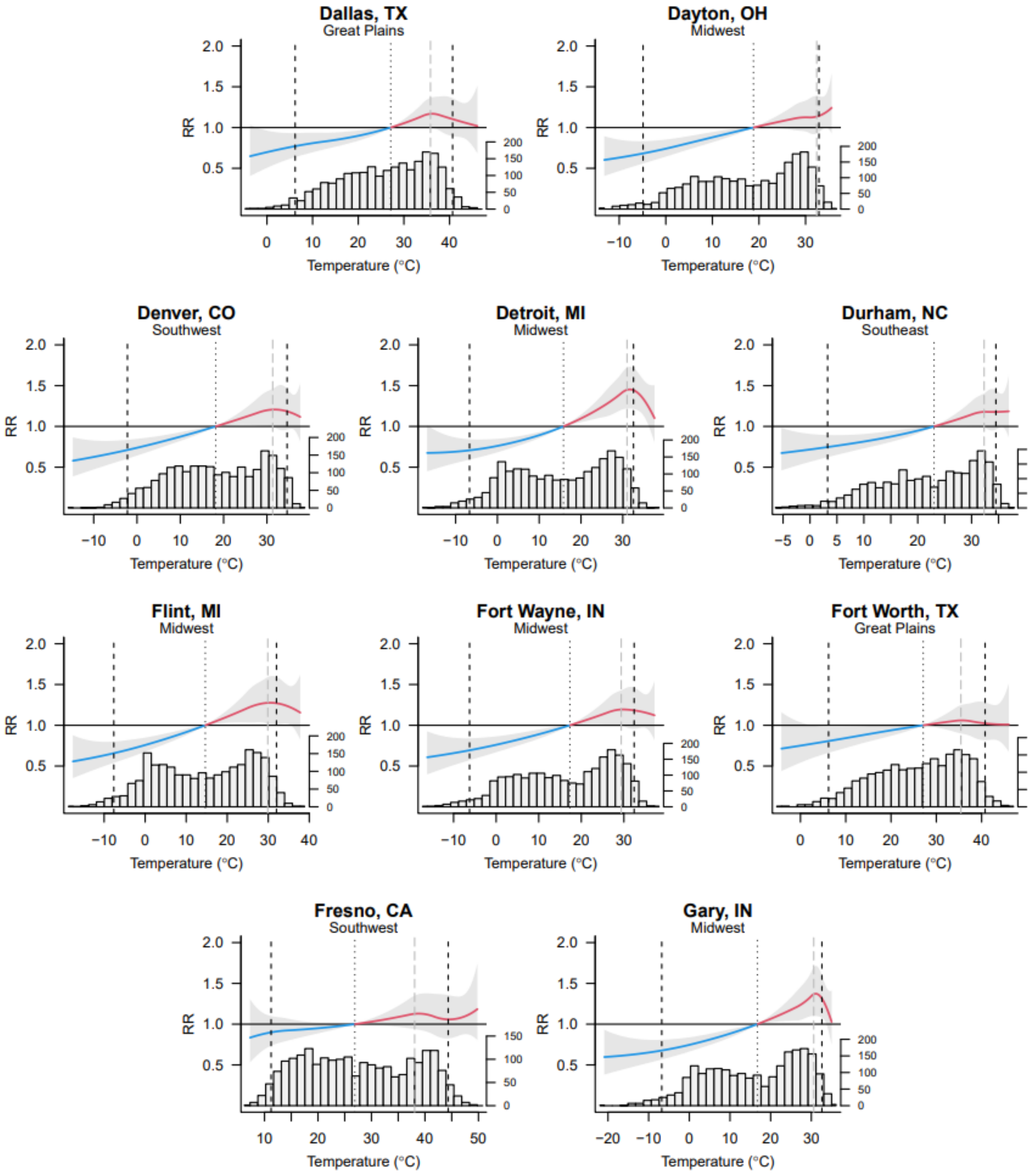

© 2022 Lyons VH et al. *JAMA Network Open*.

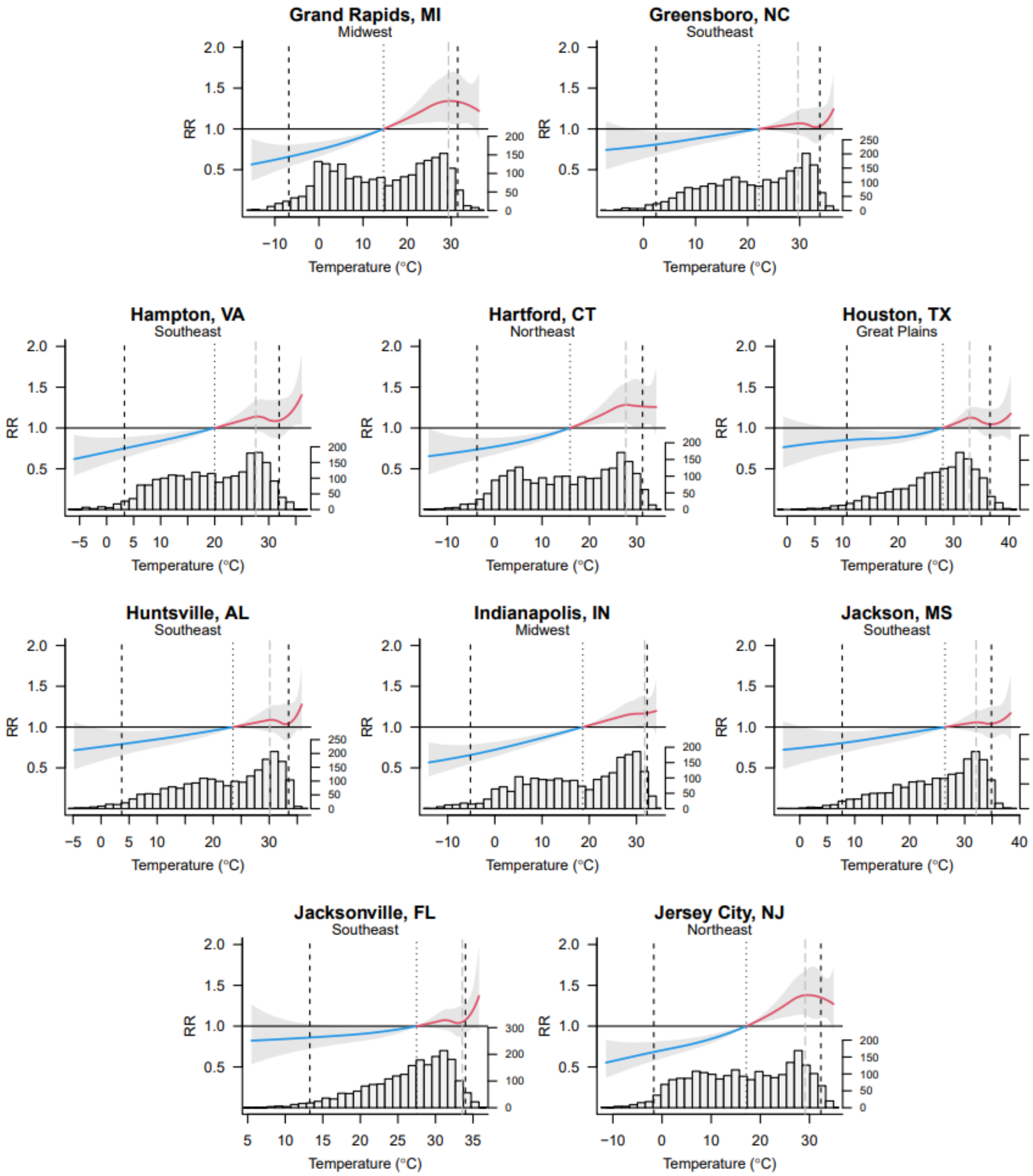

© 2022 Lyons VH et al. *JAMA Network Open*.

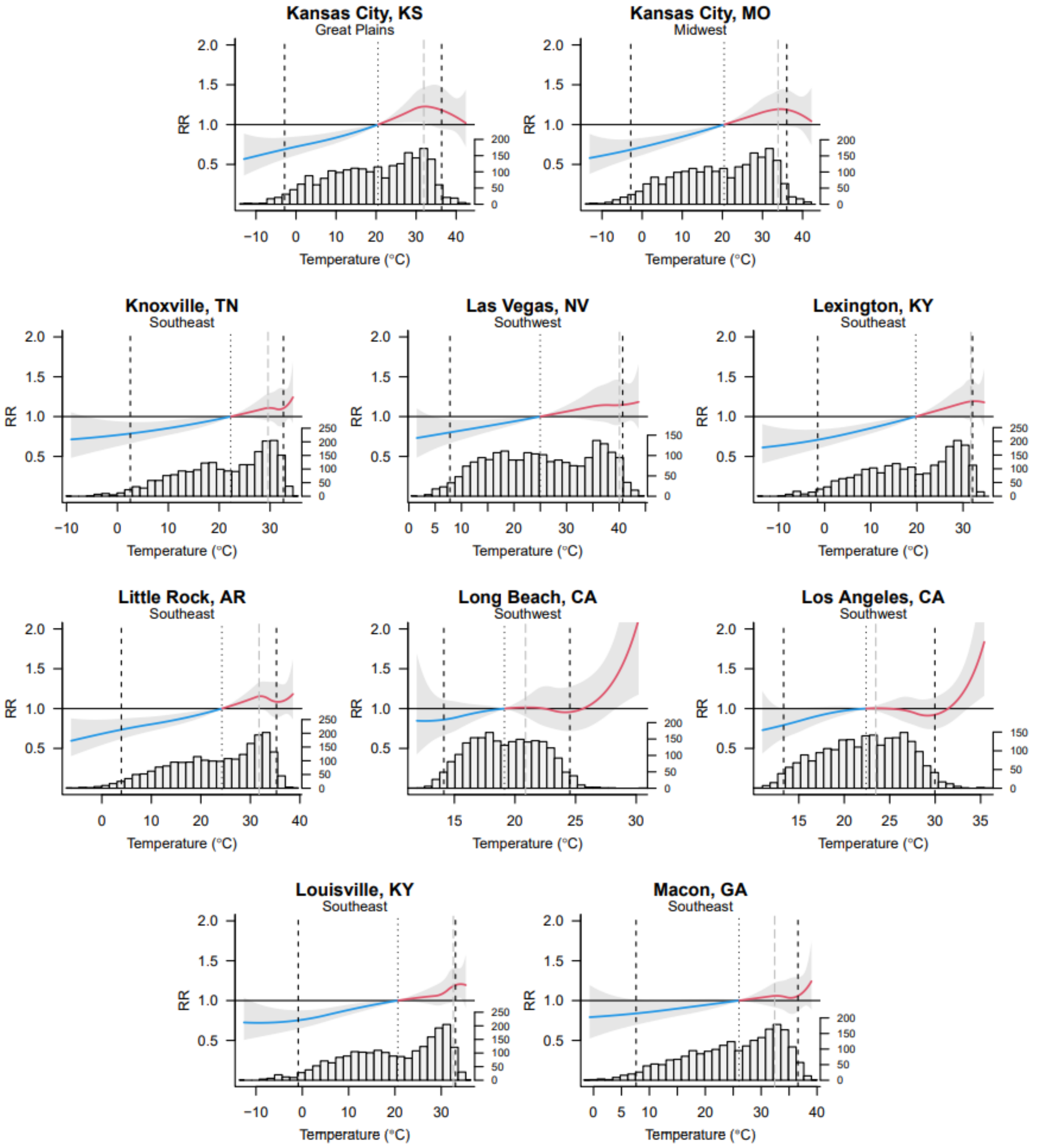

© 2022 Lyons VH et al. *JAMA Network Open*.

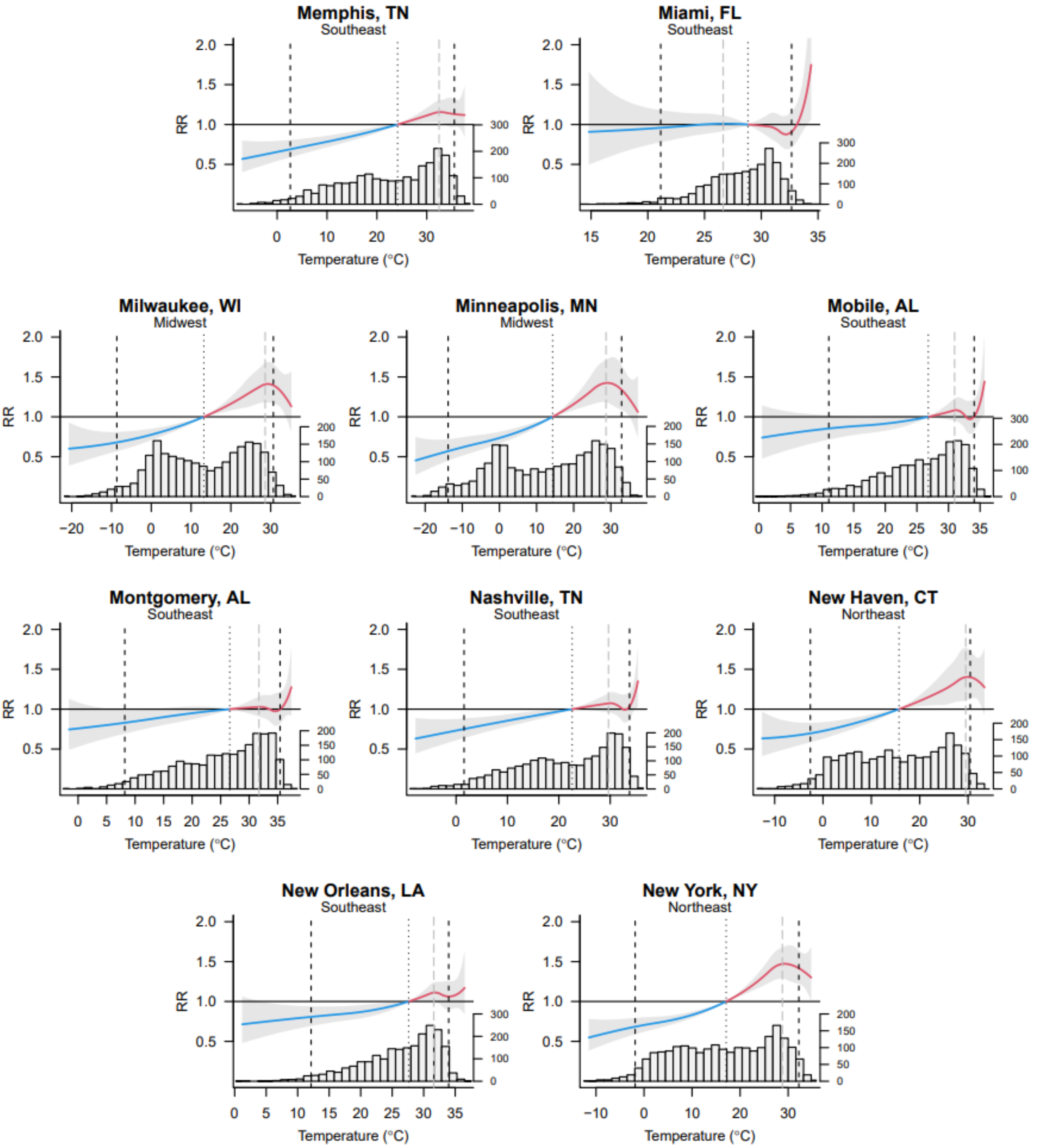

© 2022 Lyons VH et al. *JAMA Network Open*.

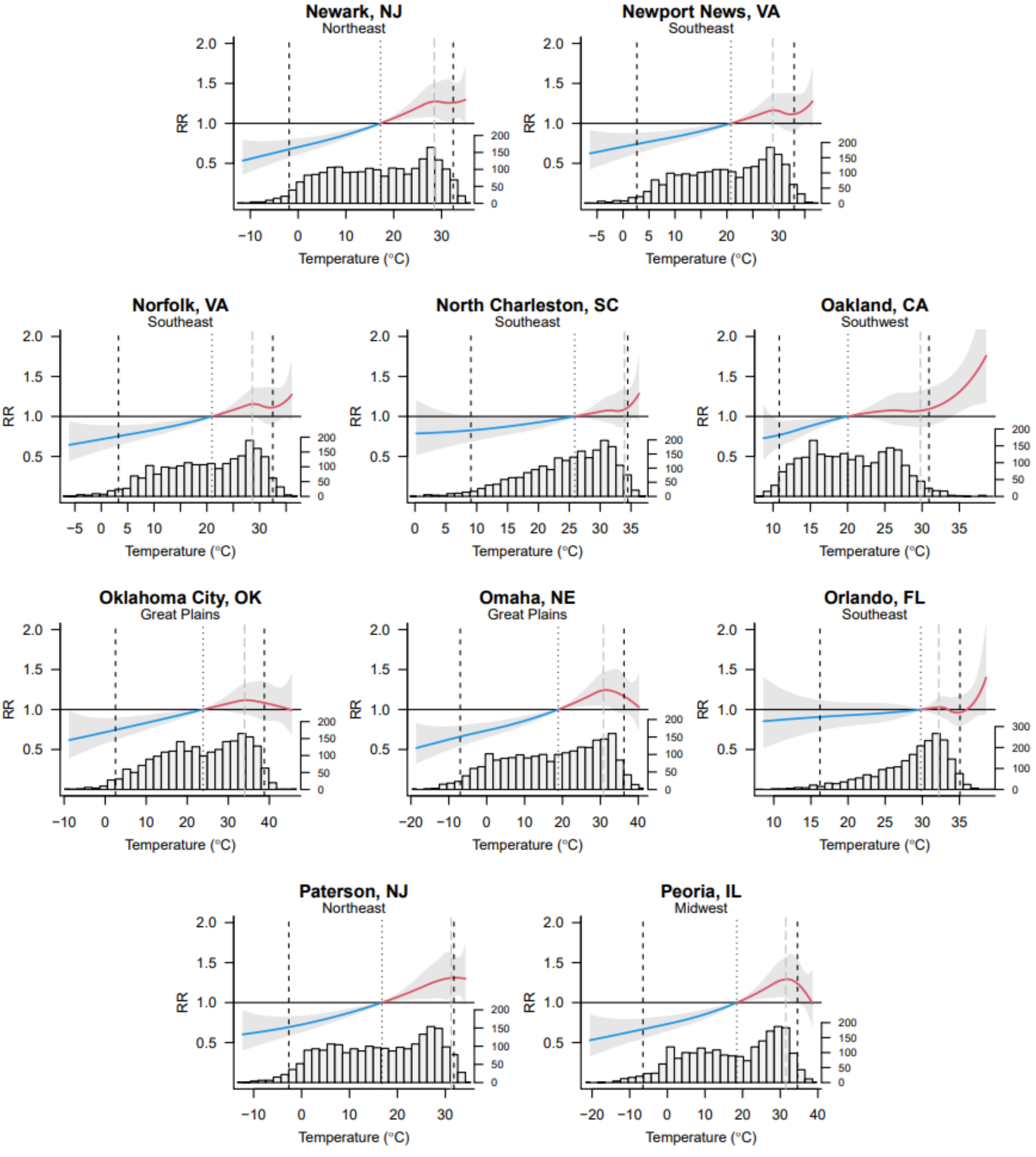

© 2022 Lyons VH et al. *JAMA Network Open*.

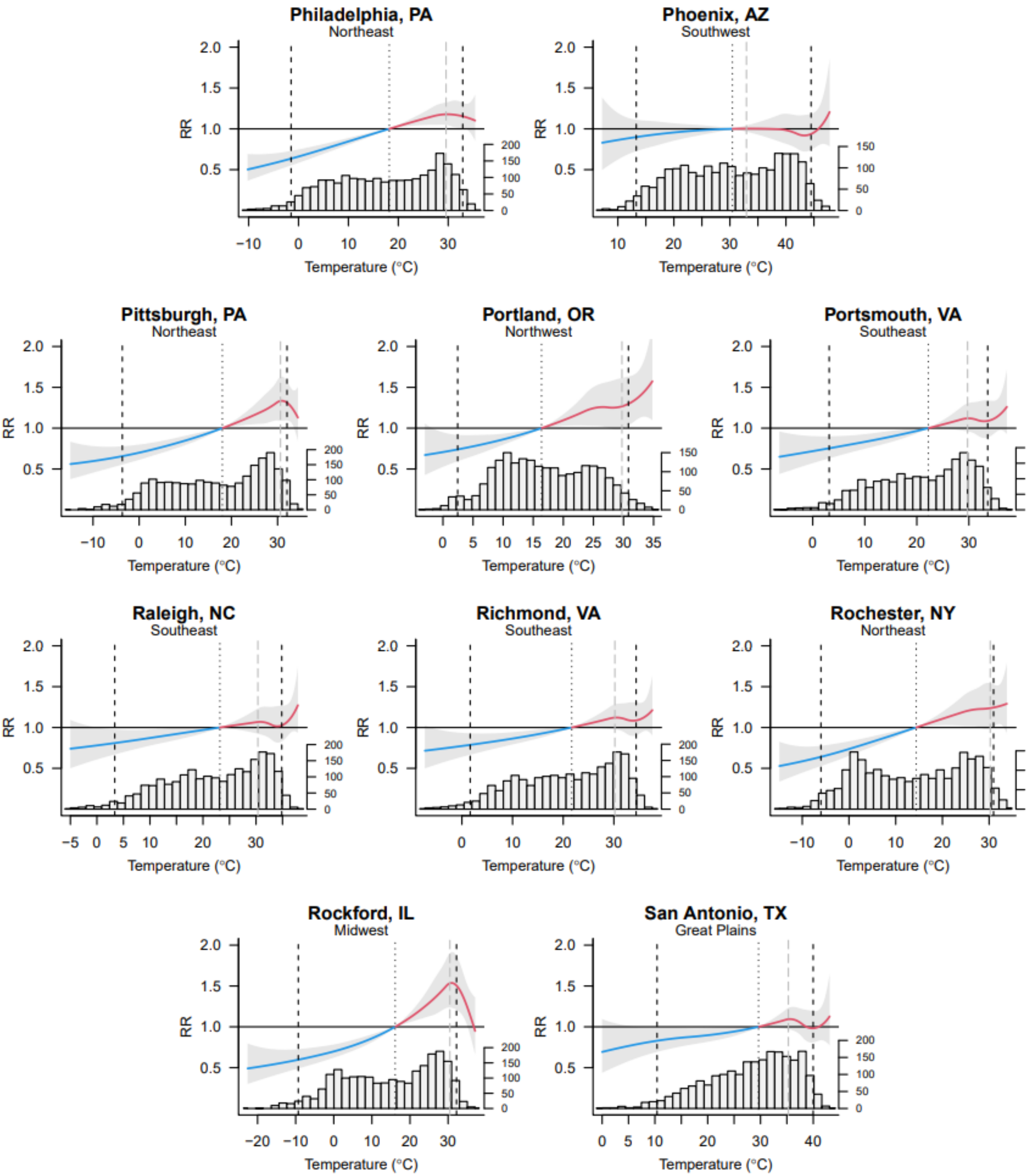

© 2022 Lyons VH et al. *JAMA Network Open*.

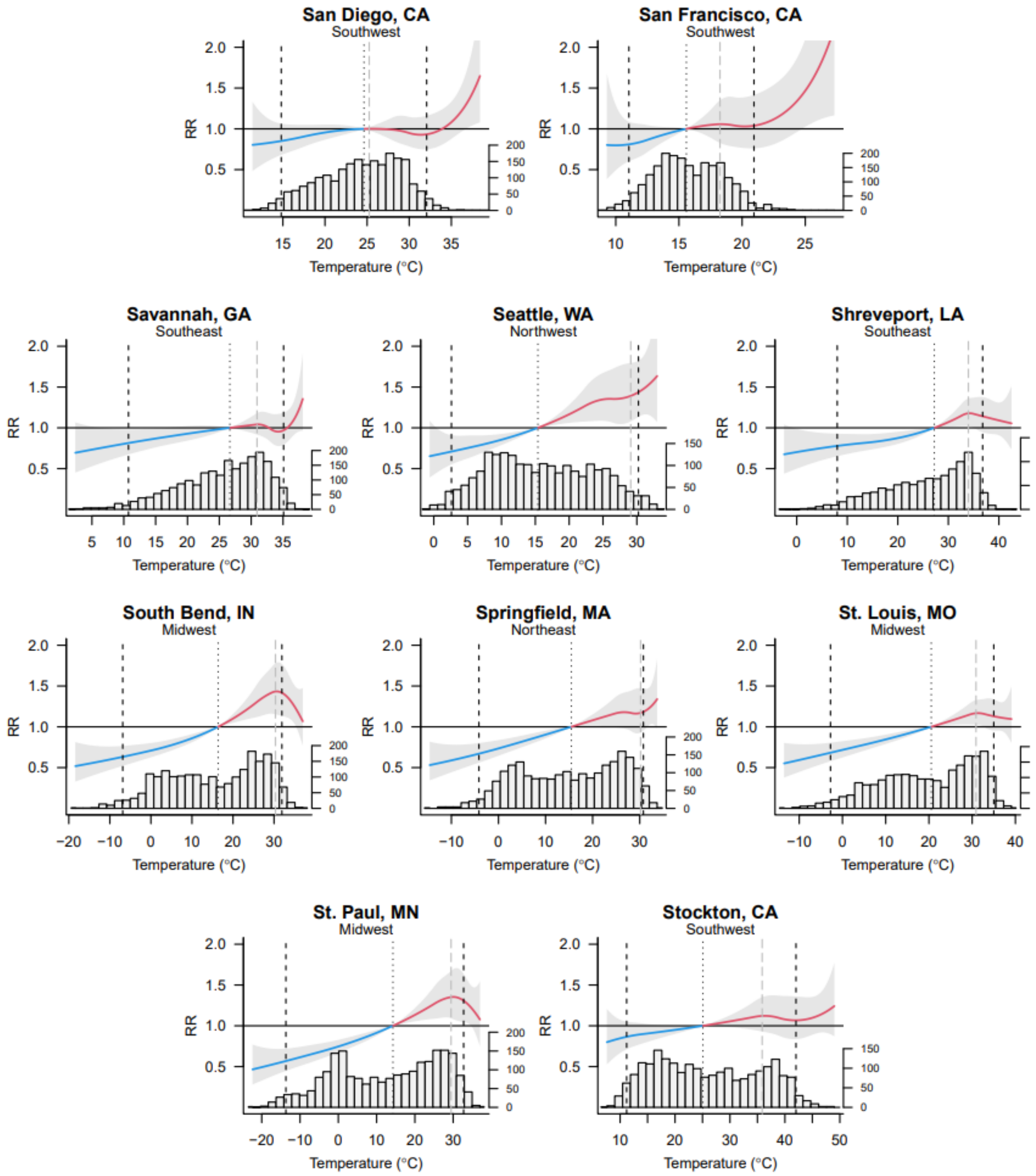

© 2022 Lyons VH et al. *JAMA Network Open*.

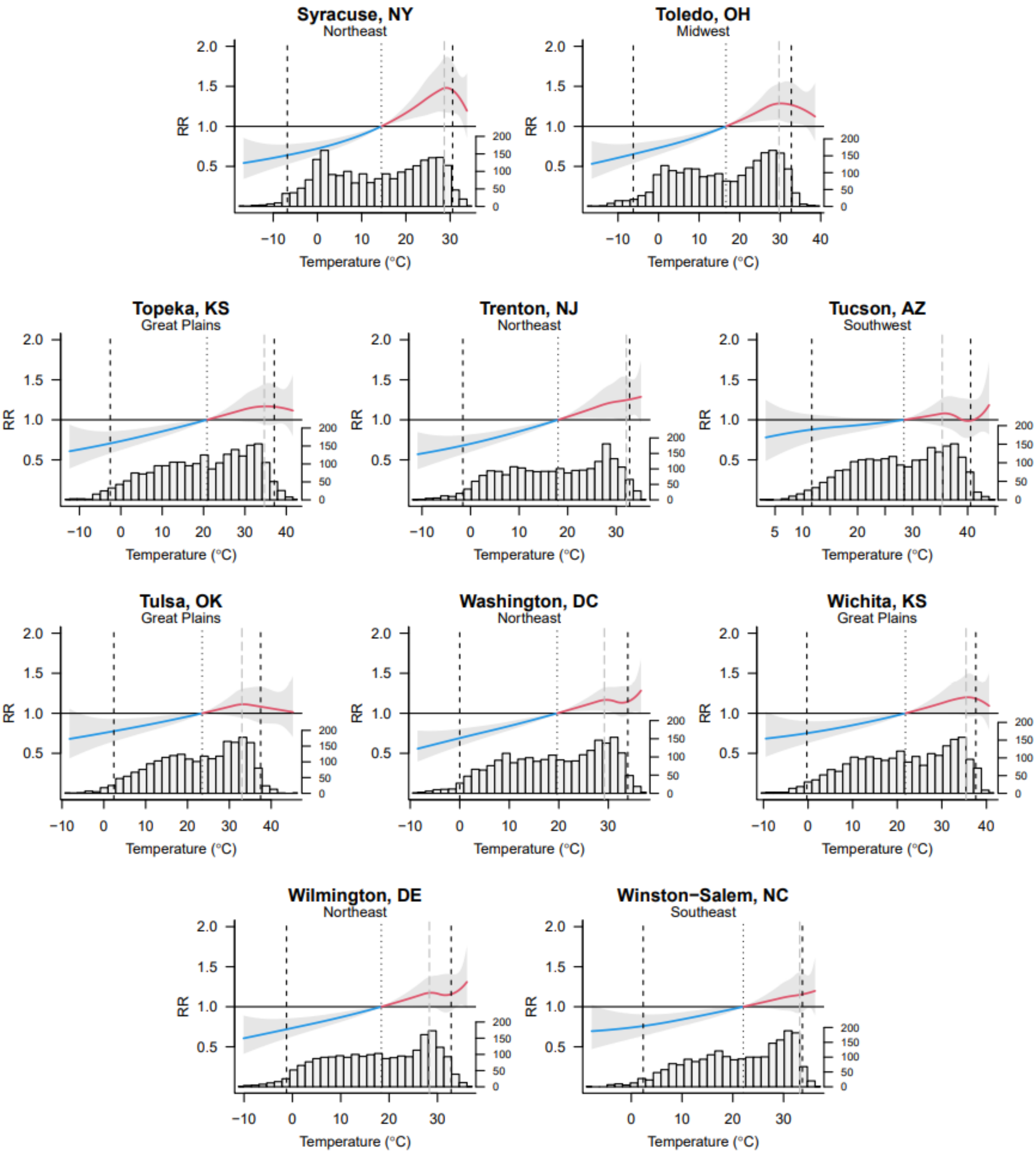

© 2022 Lyons VH et al. *JAMA Network Open*.
