# Supplementary material for: Analysis of Daily Ambient Temperature and Firearm Violence in 100 US Cities
Source: JAMA Netw Open. 2022 Dec 16;5(12):e2247207. doi: 10.1001/jamanetworkopen.2022.47207 (PMC9856408; doi:10.1001/jamanetworkopen.2022.47207)
Supplement: Supplement 2. — Data Sharing Statement [file jamanetwopen-e2247207-s002.pdf]

Data Sharing Statement

Lyons. Analysis of Daily Ambient Temperature and Firearm Violence in 100 US Cities. *JAMA Netw Open*. Published December 16, 2022. doi:10.1001/jamanetworkopen.2022.47207

Data
Data available: Yes
Data types: Data (not involving human participants)
How to access data: Data is available upon request to egause@bu.edu.
When available: With publication

Supporting Documents
Document types: Statistical/analytic code
How to access documents: We have uploaded our code to a publicly available GitHub repository found here: https://github.com/2022_Lyons_JNO_Rcode with additional details in our Appendix.
When available: With publication

Additional Information
Who can access the data: Data will be made available to researchers whose proposed use of the data has been approved.
Types of analyses: Any approved research project.
Mechanisms of data availability: After approval of a proposal and with a signed data access agreement.
